# Layering and scaling up chronic non‐communicable disease care on existing HIV care systems and acute care settings in Kenya: a cost and budget impact analysis

**DOI:** 10.1002/jia2.25496

**Published:** 2020-06-19

**Authors:** Brianna Osetinsky, Ann Mwangi, Sonak Pastakia, Marta Wilson‐Barthes, Joan Kimetto, Kimutai Rono, Jeremiah Laktabai, Omar Galárraga

**Affiliations:** ^1^ Health Services, Policy, and Practice Brown University School of Public Health Providence RI USA; ^2^ Health Systems and Policy Swiss Tropical and Public Health Institute Basel Switzerland; ^3^ Academic Model Providing Access to Healthcare (AMPATH) Eldoret Kenya; ^4^ Department of Behavioral Science School of Medicine Moi University Eldoret Kenya; ^5^ Department of Pharmacy Practice Purdue Kenya Partnership Purdue University College of Pharmacy Eldoret Kenya

**Keywords:** budget impact, chronic care, costing, modelling, integrated, non‐communicable disease, HIV

## Abstract

**Introduction:**

Like many countries in sub‐Saharan Africa, Kenya is experiencing a rapid rise in the burden of non‐communicable diseases (NCDs): NCDs now contribute to over 50% of inpatient admissions and 40% of hospital deaths in the country. The Academic Model Providing Access to Healthcare (AMPATH) Chronic Disease Management (CDM) programme builds on lessons and capacity of HIV care to deliver chronic NCD care layered into both HIV and primary care platforms to over 24,000 patients across 69 health facilities in western Kenya. We conducted a cost and budget impact analysis of scaling up the AMPATH CDM programme in western Kenya using the International Society for Pharmacoeconomics and Outcomes Research guidelines.

**Methods:**

Costs of the CDM programme for the health system were measured retrospectively for 69 AMPATH clinics from 2014 to 2018 using programmatic records and clinic schedules to assign per clinic monthly costs. We quantified the additional costs to provide NCD care above those associated with existing HIV or acute care services, including clinician, staff, training, travel and equipment costs, but do not include drugs or consumables as they would be paid by the patient. We projected the budget impact of increasing CDM coverage to 50% of the eligible population from 2021 to 2025, and compared it with the county budgets from 2019.

**Results:**

The per visit cost of providing CDM care was $10.42 (SD $2.26), with costs at facilities added to HIV clinics $1.00 (95% CI: −$2:11 to $0.11) lower than at primary care facilities. The budget impact of adding 26,765 patients from 2021 to 2025 to the CDM programme was 3,088,928 under constant percent growth, and 3,451,732 under steady‐state enrolment. Scaling up under the constant percent growth scenario resulted in 12% cost savings in the budget impact. The county programmatic CDM cost in 2025 was <1% of the county healthcare budgets from 2019.

**Conclusions:**

The budget impact of scaling up AMPATH’s CDM programme will be driven by annual growth scenarios, and facility/provider mix. By leveraging task shifting, referral systems and partnering with public and non‐profit clinics without NCD services, AMPATH’s CDM programme can provide critical NCD care to new, rural populations with minimal financial impact.

## 1 Introduction

In low‐ and middle‐ income countries (LMICs), the burden of non‐communicable diseases (NCD) is rapidly increasing due to ageing populations, lifestyle and dietary changes, rapid urbanization, and improved control of communicable diseases [1]. Of the 14 million annual premature deaths attributable to NCDs, 90% occur in LMICs where availability and use of appropriate NCD services is insufficient, especially in poorer and rural areas [2, 3]. In Kenya 14% of adults have three or more risk factors for cardiovascular disease (CVD) and 25% have hypertension or diabetes as of 2015, with models predicting increasing growth [4, 5]. The prevalence of NCDs is also rising among the HIV infected population, increasing mortality and further complicating chronic disease treatment including adherence to antiretroviral (ART) medications [6, 7]. To address this emerging epidemic, the Kenyan Ministry of Health (MOH) developed the National Strategy for the Prevention and Control of Non‐Communicable Diseases to implement efficient mobilization and utilization of resources [8]. One of the guiding principles of this strategy is integration of non‐communicable disease control into existing primary care and HIV treatment platforms, as NCD care had historically only been available in hospitals. In addition to better coordinating treatment for NCD/HIV comorbidities associated with ageing, this strategy leverages the advancements in health systems infrastructure, training, and workforce task‐shifting developed for HIV care platforms to address the systemic deficits in chronic disease care [9].

An HIV/NCD modelling study in Kenya estimated that integrated HIV and NCDs could avert more than 43,000 CVD‐related deaths over 15 years but that the cost required to fully scale‐up the intervention would require a 12% increase in Kenya’s total health budget [10]. Another study in Uganda also demonstrated cost‐effectiveness of integrated HIV/NCD care but noted that adding this integration would account for 4% of the national HIV budget [11].

Layering chronic disease care into existing HIV programmes, and further expanding that integration within effective primary care platforms, strengthens the capacity of the public‐sector healthcare system to treat NCDs under resource constraints [12]. In western Kenya, the Academic Model Providing Access to Healthcare (AMPATH) chronic NCD care programme has been integrated with existing HIV and primary care programmes and shown to be effective for improving retention in care and hypertension control among HIV positive patients, which addresses an important gap in NCD care delivery for this population [13, 14, 15, 16]. The flexibility of this integrated model allows for programme expansion based on local needs and existing health system capacity. Policy changes that aim to finance public programmes and increase enrolment in and reimbursements for the National Health Insurance Fund (NHIF) require a clear understanding of the affordability of NCD treatment programmes. To inform the affordability of scaling up chronic NCD care within AMPATH’s existing HIV and primary care programme in western Kenya, we measured the cost of the programme from a health system perspective from 2014 to 2018 and modelled the budget impact of increasing programme coverage from 2021 to 2025 (see Research Agenda).

## 2 Methods

### 2.1 Study design

We conducted a retrospective costing and applied the International Society for Pharmacoeconomics and Outcomes Research (ISPOR) framework to perform the Budget Impact Analysis (BIA [17]. We measured the incremental costs to the health system of layering the CDM programme into existing outpatient public and non‐profit primary care and HIV care platforms in western Kenya. We constructed a static deterministic model to quantify the budget impact of increasing the CDM programme to achieve the health systems NCD treatment target of drug therapy and counselling coverage of 50% of the eligible population [18], which is one of nine voluntary global targets set by the WHO’s Global Monitoring Framework for NCDs and adapted for the Kenyan National Strategy for The Prevention And Control of NCDs.

### 2.2 Study setting

In western Kenya, the AMPATH provides comprehensive HIV care for more than 140,000 HIV‐infected patients throughout a catchment area of over 3.5 million people. In collaboration with the MOH, AMPATH formed its Chronic Disease Management (CDM) programme with the goal of leveraging existing health delivery platforms to expand treatment capacity.

Costing was conducted for all 69 AMPATH‐affiliated health facilities providing chronic NCD care, including 23 HIV care facilities. CDM care is delivered in 37 dispensaries, 16 health centres, 15 primary hospitals, and the Moi Teaching and Referral Hospital (MTRH) (Tables S1 and S2).

The CDM programme draws from the model of task‐shifting and community partnerships build upon the capacity and willingness of established healthcare delivery systems [19]. In addition to training nurses who are located at the CDM clinics, clinical officers, pharmacists and physicians are based out of regional hospitals, and travel throughout the county to dispensaries, health centres and primary care hospitals for regular NCD visit days, with frequency driven by patient demand and clinician workloads [20, 21, 22, 23]. More complex patients are referred to higher levels of care as needed, and the programmatic staff and AMPATH Medical Records System (AMRS) provides consistent chronic disease tracking. The CDM programme was initially delivered on top of AMPATH’s HIV care but has since expanded across other existing MOH or not‐for‐profit facilities, and now accepts NCD patients regardless of HIV status [9]. Partnering with established health facilities enables the CDM programme to provide comprehensive care without having to create new facilities. This study was conducted in the counties of Bungoma, Busia, Kisumu, Nandi, Trans Nzoia and Uasin Gishu, where AMPATH has established local partnerships and variable CDM program penetration.

### 2.3 Ethics approval

This retrospective costing and modelling study used de‐identified data from the AMRS, which does not require individual informed consent. The Institutional Research and Ethics Committee at the Moi University School of Medicine and the Institutional Review Board at Brown University approved use of these de‐identified data and waived informed consent requirements.

### 2.4 Input data and sources

We quantified the additional costs of the CDM programme outside of those incurred by the existing primary and HIV care programmes, including training, clinicians, programme and data management staff, medical records though the AMRS and testing/monitoring equipment. Consumables and medications are paid for by patients and were not included in this analysis.

Monthly programme costs from 2014 to 2018 were collected via key informant interviews with programme officers and clinicians, CDM programme records, and AMPATH employee salary scales and standard operating procedures. Facility costs were assigned using monthly patient visits and the CDM programme’s monthly schedule, which captured variation in visit frequency and clinical staff over time (full cost categorizations and facility attributes are provide in Tables S5,S6,S7,S8,S9,S10). Personnel costs included clinician salary, training costs, and programmatic staff salary. Travel costs are incurred when clinical staff travel to facilities from the referral hospitals in each county or MTRH. They are calculated using round trip distance to the geocoded facility location multiplied by the cost per kilometre provided by the AMPATH Care Standard Operating Procedures for Clinical Travel. Costs of equipment used for diagnosis and monitoring were based on market prices and annualized using key informant reports of equipment lifetime under standard use in the programme [24]. All facilities used electronic blood pressure cuffs to assess blood pressure, and glucometer for blood glucose, and either handheld or bench‐top laboratory instruments for A1C measurement. Monthly patient visits by clinic were derived from the AMRS reports.

#### 2.4.1 Cost analysis

All costs were standardized to AMPATH’s 2018 payment schedules and standard operating procedures, and all calculations used the average 2018 exchange rate of 101.35 Kenya shillings to one United States dollar [25]. Costs analyses include a description of monthly costs per facility and annual mean cost of facilities and patient visits. We assessed the differences in costs between the CDM facilities added to the HIV platform compared to CDM facilities added to a primary care platform controlling for differences in facility levels, transport costs, and time trends, and a non‐linear transformation of number of facility visits to account for economies of scale using the following formula:

$$\begin{array}{cc} \text{Cost}= & \beta_{0}+\beta_{1}\text{HIV}\;\text{Clinic}+\beta_{2}\text{Facility}\;\text{Patient}\;\text{Visits}\;\\ & +\beta_{3}\log\;\text{Facility}\;\text{Patient}\;\text{Visits}+\beta_{4}\;\text{Facility}\;\text{Level}+\beta_{5}\;\text{Trasport}+\beta_{6}\;\text{Time}\;\text{Trend} \end{array}$$

### 2.5 Budget impact model

In the model we included the following incremental costs: clinic visits, clinical staff salary, programmatic staff salary, training, and travel, as well as incremental equipment costs by clinic. We used normative CDM treatment guidelines to transform the incremental patient visit costs to the annual total patient costs based on management plans reflecting the treatment complexity and attrition among the CDM population (see Tables S5,S6,S7,S8,S9,S10 for more detail) [26].

#### 2.5.1 Target population

**Figure 1:**
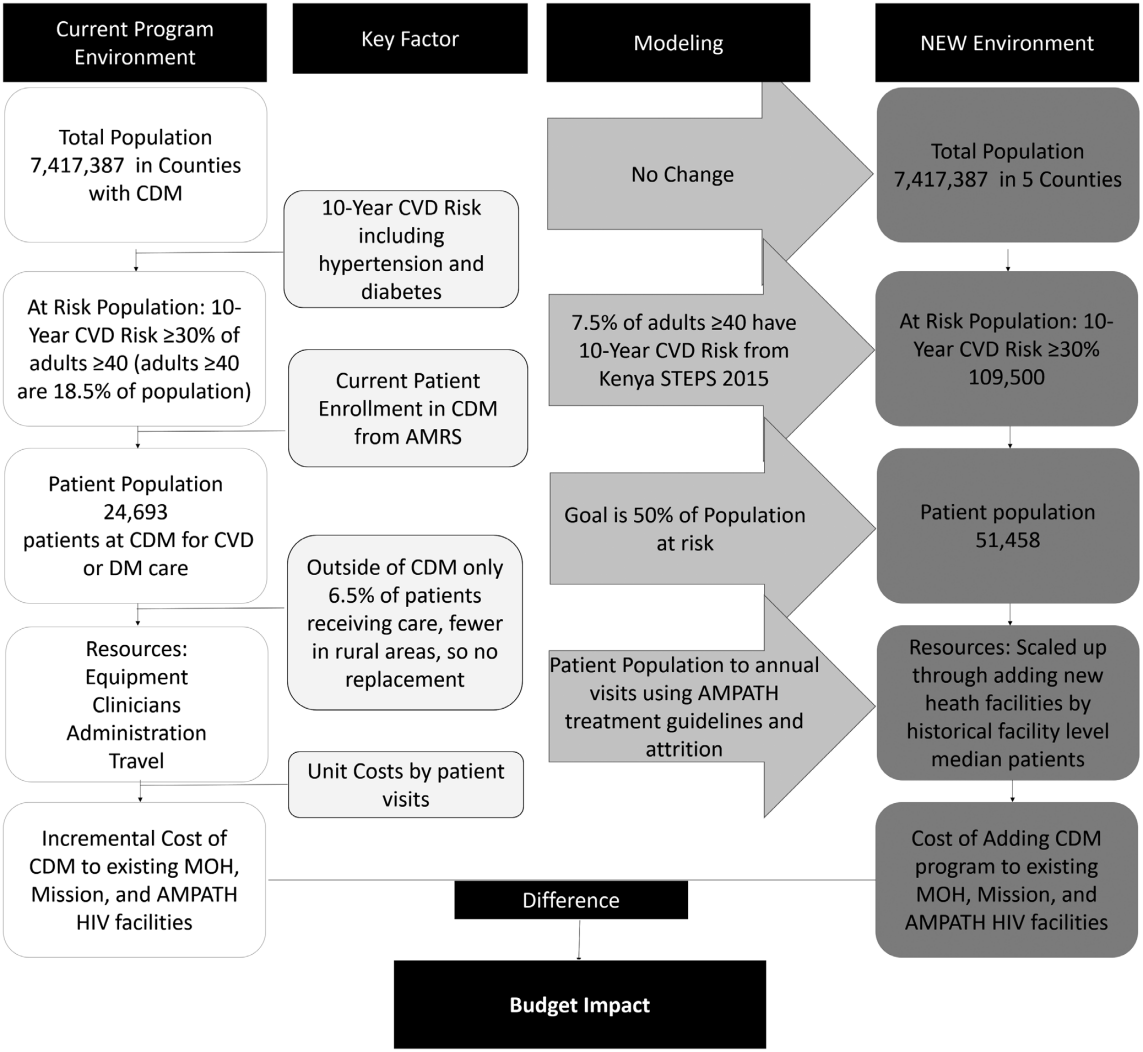
Budget impact schematic. This analysis models the change in total target population, increasing the number of CDM facilities, and predicted increase in NCD prevalence. Adapted from ISPOR Budget Impact‐Principals of Good Practice Taskforce [17].

The BIA estimates the cost of scaling up CVD care including hypertension and diabetes treatment to reach 50% of the eligible population by 2025 for the six counties where AMPATH is partnered with the public health system (Figure 1). The eligible population for the CVD care target are people over 40 with a 10‐year CVD risk profile ≥30% or CVD, which is 7.5% of the population ≥40 [5]. Of the six counties, two had achieved the target, and CDM did not need to be further scaled in those counties. The BIA assumptions are provided in Appendix Table S11.

#### 2.5.2 Time horizon

We modelled growth over a five‐year time horizon from 2021 to 2025 to align with the 2025 target achievement date of the WHO’s Global Monitoring Framework for NCDs and Kenya’s National NCD Strategy.

#### 2.5.3 Scenario analysis

We modelled the scale‐up needed to achieve these the targeted number of patients within five years per country in two ways: steady‐state enrolment where the same number of patients are added annually, and a constant growth percentage where the number of new patients is derived as a percent of the prior year’s total patients.

#### 2.5.4 Budget holder

The budget holder is the county government that allocates health funding and care delivery. However, since most health policy and financing is at the national level we also present results for the total programme. As availability of NCD care is very low for this patient population outside of the CDM programme, we did not include a replacement scenario where patients can receive NCD care elsewhere. Due to the short time frame used in the BIA and the need to budget spending in each year, we do not include discounting.

#### 2.5.5 Cost inputs

The cost is quantified separately for each facility level as the largest per patient cost variation was across facility levels. To account for patient‐volume economies of scale and limits of individual clinics, the median monthly patient visits for each facility level was used for the incremental cost‐per‐patient, and to quantify the number of facilities and therefore the amount of addition equipment needed. Equipment costs are included in the year they would be purchased. The proportion of the total additional patients allocated to each facility level is based on historical trends in proportion of patients in each level. The BIA is reported separately for each county and for the whole programme.

### 2.6 Sensitivity analyses

We conducted multivariate and univariate deterministic sensitivity analysis of the costs inputs, median patients per facility, and two alternative distributions of new patients across the facility levels, one prioritizing growth in primary care facilities, and another demonstrating equal growth by facility type. We conducted sensitivity analysis on the travel cost using the interquartile range of the whole programme because counties with fewer programmes may be more closely grouped together and would not accurately reflect programme expansion throughout the entire county.

## 3 Results

**Figure 2:**
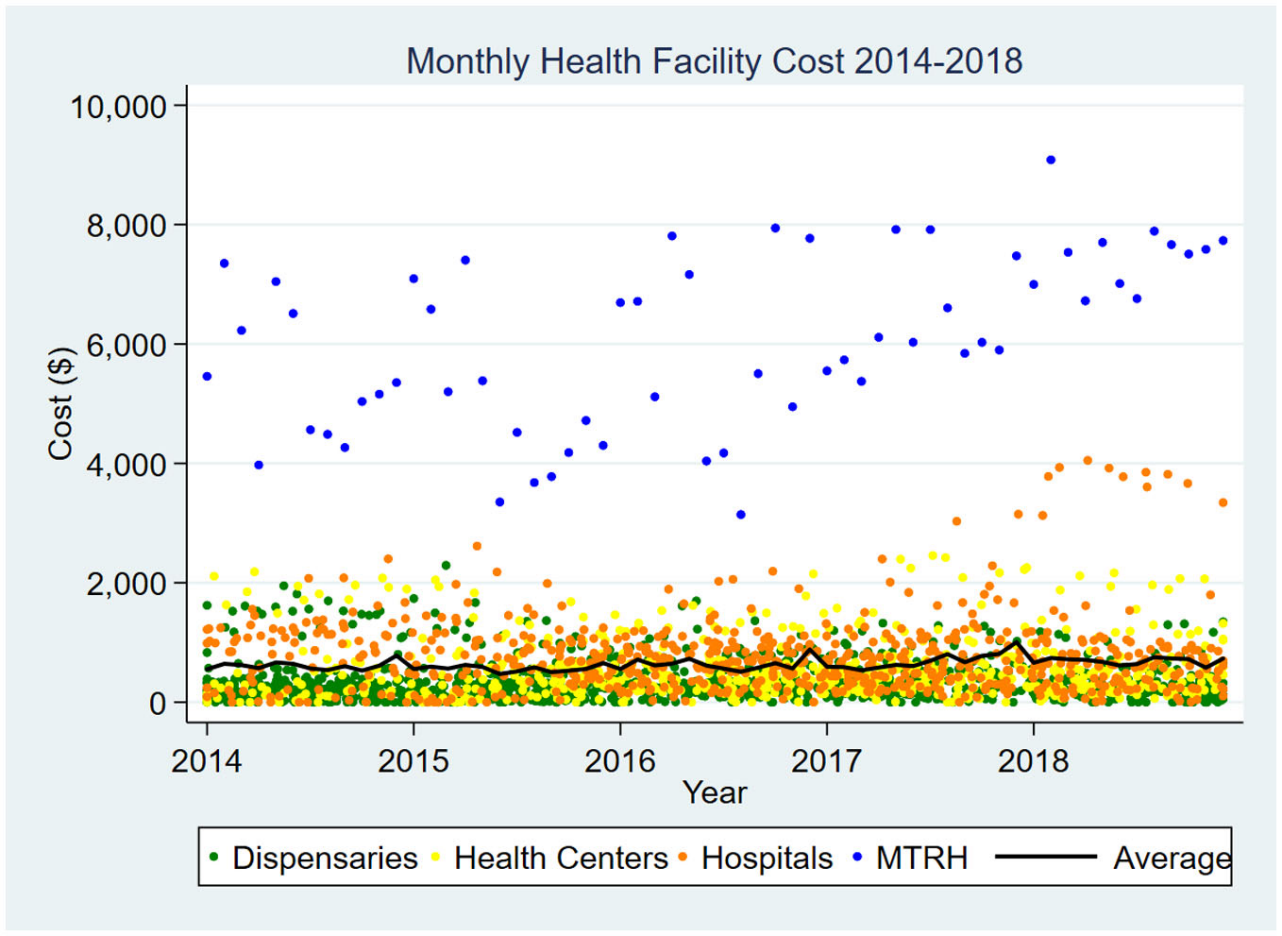
Monthly Programmatic Costs by Facility, and Average Total Cost per Month from January 2014 to 2018.

Figure 2 provides the monthly costs among active facilities from 2014 to 2018. While the average costs are stable over time, the variation reflects differences in provider visits and monthly patient visits. MTRH is a clear outlier with monthly costs>$3,500 across the study period. There are also distinct distributions in costs by facility type, despite more overlap across the levels of the health system. Because the costing is reflective of a dynamic treatment program that is adaptive to patient demand and programme capacity, the number of facilities and cost of each varies throughout the period of study.

Due to this variation, we separate the levels of care and present the mean and standard deviation (SD) of annual and monthly costs by facility and patient visits in Table 1. The average annual facility cost of CDM varied across the four levels from $3,862 (SD: 373) per dispensary to $72,671 (SD: 10,404) at MTRH. For each level of care, the cost of clinicians and administrative costs made up the largest proportion of total costs. The average total cost per patient‐visit ranged from $8.21 (SD: $2.21) at primary hospitals to $18.42 (SD:$3.61) at MTRH. Per facility annualized equipment costs varied by level from $167 (SD 35) to $283 (SD: $39). MTRH had a considerably higher average clinician cost at $64,073 (SD$10,404). This is unsurprising given that MTRH functions as both a Cardiac Center of Excellence and a referral hospital, seeing both the largest volume of and most complex patients who require a higher proportion of clinical specialists. With the exception of the referral hospital (MTRH), travel costs by visit ranged from $0.80 to $2.99 across all levels of care. Staff costs were evenly distributed by patient visits, with annual costs of $805 per dispensary, $2,002 per health centre, $3,662 per primary hospital and $18,314 at MTRH.

**Table 1:** Facility annual cost and cost per patient visit by cost categories in US($) means and standard deviations (SD), 2014 to 2018

Cost type	Dispensaries		Health centres		Primary hospital		Referral hospital		Program average	
	Facility (SD)	Patient visit (SD)	Facility (SD)	Patient visit (SD)	Facility (SD)	Patient visit (SD)	Facility (SD)	Patient visit (SD)	Facility (SD)	Patient visit (SD)
Clinicians	1490 (88)	4.43 (0.99)	2356 (361)	3.32 (0.74)	2,947 (163)	2.60 (0.74)	64,073 (19,311)	17.11 (7.17)	301 (40)	5.20 (1.98)
Staff	1548 (278)	4.55 (1.21)	3138 (410)	4.46 (1.21)	5009 (614)	4.49 (1.23)	18,314 (3300)	4.65 (1.17)	270 (21)	4.5 (1.21)
Travel	1068 (449)	2.99 (0.87)	945 (163)	1.32 (0.24)	898 (78)	0.80 (0.19)	0 (0)	0 (0)	79 (20)	1.28 (0.27)
Equipment	167 (35)	0.48 (0.05)	326 (58)	0.46 (0.12)	321 (43)	0.29 (0.08)	283 (39)	0.07 (0.02)	20 (3)	0.33 (0.07)
Total	3862 (373)	11.35 (2.09)	4,268 (404)	9.38 (1.97)	6,9116 (685)	8.21 (2.21)	72,671 (10,404)	18.42 (3.61)	629 (48)	10.42 (2.26)
Volume^a^	19 (5)	29 (38)	10 (3)	63 (69)	11 (3)	101 (134)	1 (0)	341 (114)	42 (8)	63 (14)

^a^ Average number of active facilities by month, and average number of patients by facility, with standard deviations.

Table 2 presents the difference in patient visit costs for CDM program integration within the HIV care platform compared to the primary care platform. Controlling for patient visits, health care level, transportation costs, and date (month and year), NCD care provision within the HIV care platform was $1 (95% confidence interval (CI), −2.11 to 0.11) less expensive per patient visit compared to care provision added to the primary care platform.

**Table 2:** Per patient visit costs by care delivery platform

	Coef.	P> t	[95% Conf. interval]
HIV care platform	−1.00	0.079	(−2.11, 0.12)
Monthly patient visits	0.012	0.002	(0.004, 0.019)
Log (monthly patient visits)	−5.71	<0.001	(−6.22, −5.19)
Facility level			
Health centre	−0.57	0.353	(−1.77, 0.63)
Hospital	0.48	0.455	(−0.77, 1.73)
Referral hospital	20.27	<0.001	(16.68, 23.87)
Date (month/year)	−0.015	0.317	(−0.044, 0.014)
Transportation costs	0.069	<0.001	(0.061, 0.077)
_cons	38.99	<0.001	(19.56, 58.41)

**Figure 3:**
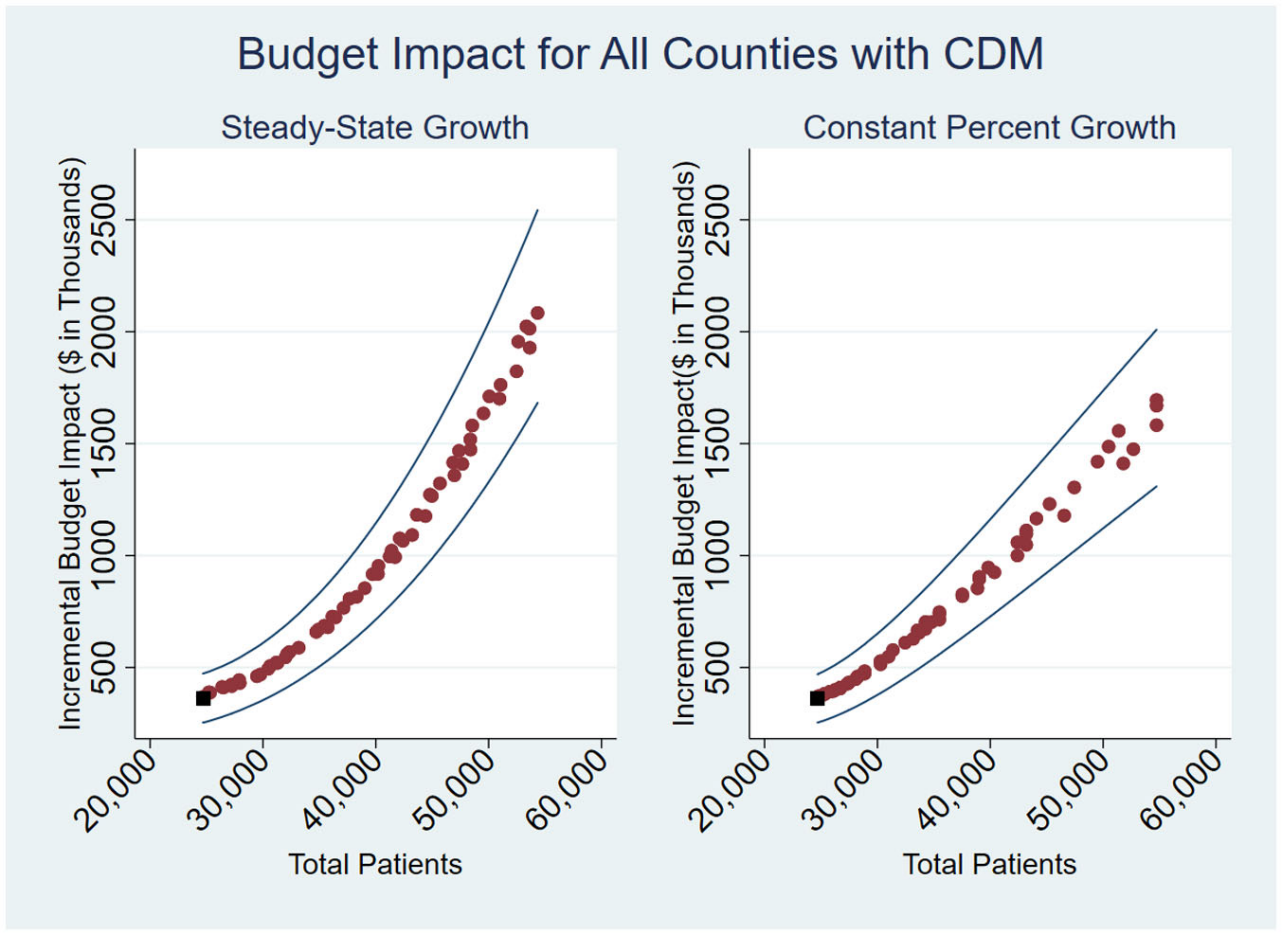
Budget Impact of Scaling up CDM to the treatment targets under two scale‐up models: Incremental costs of the additional patients from 2021 to 2025.

Under the constant percent growth patient enrolment scenario, the incremental five‐year budget impact of scaling up CDM across all CDM counties was $3,088,970, while under the steady state scale‐up scenario the incremental five‐year budget impact was $3,451,732, with a total number of 26,765 new patients enrolled (Table 3). In Bungoma county, the budget impact under the percent growth scale‐up and steady‐state scenarios is $539,820, and $722,798 respectively, a 34% difference. In Kisumu county, the incremental five‐year budget impact under the percent growth scale‐up is $287,977 and $390,183 under the steady state scale‐up, representing a 35% difference. In Nandi county, the incremental five‐year budget impact under the percent growth scale‐up and steady state is $2,274,405 and 336,493 respectively, a 23% difference. In Trans Nzoia county, the incremental five‐year budget impact under the percent growth scale‐up is $187,041 and $202,572 under the steady state scale‐up scenario. Savings from the percent growth scale‐up scenario in Trans Nzoia is minimal at only 8%. The differences in costs under the two scale‐up scenarios are shown for the whole programme in Figure 3, which presents the incremental cost of scale‐up by total number of covered patients under all scenarios including sensitivity analysis.

**Table 3:** Budget Impact of CDM Scale‐Up from 2021 to 2025 in US ($)

	Newly enrolled patients	Steady state patient enrolment		Percent growth target		Saving from using percent scale up, %
		Mean	(Min: Max)	Mean	(Min: Max)	
Bungoma	12,834	722,798	(556,910: 861,194)	539,820	(439,830: 642,963)	34
Kisumu	7,578	390,183	(349,735: 510,658)	287,977	(251,687: 367,500)	3
Nandi	5,861	336,493	(271,193: 395,645)	274,405	(222,323: 324,735)	23
Trans Nzoia	3,572	202,573	(165,079: 240,893)	187,041	(152,491: 222,585)	8
Total	26,765	3,451,732	(2,600,555: 4,341,799)	3,088,928	(2,323,970: 3,891,191)	12

Table 4 presents (1) the 2019 annual healthcare budget for the six counties where AMPATH operates the CDM programme, and (2) the projected total program budget for the most expensive program year for the modelled time horizon (year 2025). The projected per county CDM budget also includes care costs for patients enrolled prior to the scale‐up, which allows for a more comprehensive assessment of the proportion of the annual budget that would be needed to provide CDM care to 50% of the eligible population by 2025. The proportion of annual county budget cost is less than 0.9% in every county and scale‐up scenario.

**Table 4:** County level annual budget and proportion of budget used by CDM in 2025 US($)

	Annual Healthcare Budget (2019)	Steady State Patient Enrolment 2025 CDM Budget	Percent Growth Target 2025 CDM Budget	Proportion of Healthcare Budget, %
Bungoma	30,862,211	263,006	268,323	0.4
Busia	16,908,129	104,881	104,881	0.6
Kisumu	31,039,241	141,750	150,994	0.2
Nandi	19,132,221	122,278	124,568	0.3
Trans Nzoia	21,672,952	110,654	110,715	0.3
Uasin Gishu	20,764,446	163,892	163,892	0.8

### 3.1 Sensitivity analysis

Varying the distribution of new patient enrolment across facility types led to limited variation in budget impact (Tables S16,S17,S18,S19,S20,S21). The most expensive distribution was more heavily weighted towards the dispensaries and health centres, while the least expensive option was the one based on historical expansion distribution across facility type. The budget impact using the maximum salaries was between 44% and 48% higher than the budget impact at the lowest level of salaries. The total budget impact of varying the transport costs was minimal (Tables S22,S23).

## 4 Discussion

To the best of our knowledge, this is the first study to estimate the health system delivery costs for a public NCD chronic care program in western Kenya integrated into both HIV and primary care platforms, and the first to provide the public health budget impact of scaling up integrated CDM in the region, which results in several policy implications.

While salary for clinicians and programmatic staff were the largest costs, task shifting between clinician levels to increase care coverage while still offering comprehensive care for complex cases reduces costs [9, 27, 28, 29]. However, human resource constraints may limit proposed scale‐up, and needs to be monitored using the CDM needs‐based health workforce assessment that can approximate the gaps in human resources as the program grows [26].

The average per patient visit cost was $10.42, and adding CDM to the HIV care platform cost $1.00 less than adding the same CDM services to the primary care platform. However, these findings were not statistically significant so should be considered with caution during program policy. The number of patient visits, facility level and travel demands are different for more remote HIV clinics compared to central hospitals and health centres. Though economies of scale at hospitals would result in a lower overall program budget for these facilities, a key benefit of the CDM programme is its ability to improve access to chronic care in underserved and rural areas. Thus, decision‐makers will need to balance efficient care delivery with investments to improve access to care for remote populations.

The budget projections were sensitive to the scale‐up model, with average five‐year cost savings of 12% if using scaling up with fixed percent growth instead of steady‐state enrolment. The model was also sensitive to a lesser extent to the distribution of patient enrolment across facility types, which allows for selection of a lower budget option despite rising patient‐visits. While the growth in number of patients over five years is large, the evidence from Busia and Uasin Gishu demonstrates that both rapid programme growth and achievement of the target population coverage are possible. The total annual CDM budget of the fixed percent growth model in 2025, which is the most expensive year of the programme, was very small compared to the total budget. While healthcare financing can be variable, county health budgets have increased by more than 1% every year since 2015. This signifies that the count budgets can support CDM me growth without detracting from existing services [30].

Our study has some limitations. We did not explicitly model changes in testing, linkage to care, or changing prevalence of HIV or NCDs. Using set growth rates we are instead able to project easily interpretable estimates of the budget impact, and our approach is consistent with the ISPOR guidelines which prefer static models for shorter timelines [17]. We also did not account for health system investments to improve pharmacy access or address medication stock outs [31]. In some cases the CDM programme operates concurrently with a Revolving Funds Pharmacy (RFP) model which uses revenue generated from mediation sales to sustainably resupply medications, and is used as a backup in the event of a stock‐out at the MoH facility or in the absence of an MoH pharmacy [32]. After the initial startup costs for purchasing medications and hiring pharmacists, the RFP model is largely self‐sustaining [21].

AMPATH’s CDM programme has lower average per visit costs compared to other public programs that offer chronic NCD care in Kenya, and substantially lower costs than hospitalizations related to untreated NCDs [33, 34]. This difference should not disqualify the generalizability of our findings and recommends the expansion of the CDM programme. A challenge in shaping health policy in Kenya is the lack of available evidence indicating that translation of global NCD recommendations is successful in the local context [35]. Certain costs included in this study such as the cost of travel specific to AMPATH clinics, and the differences in provider and staff pay grades compared to the public sector may result in higher total and per patient costs. Therefore, our budget projections can be seen as the higher end of the BIA estimations. The per capita budgets for the counties included in this study are consistent with other counties, which indicates that the low budget impact observed in our projections can be expected to be similar for other countries. AMPATH’s CDM programme largely serves rural and peri‐urban clinics, and expansion of this program is relevant for the 73% of the national population that reside in Kenya’s rural areas. For counties where AMPATH does not have a presence, additional costs for electronic medical records systems will need to be considered in addition to the costs for technicians and data teams included in our analyses [36].

## 5 Conclusions

By relying on task‐shifting and diverse facility partnerships, the integrated CDM programme can increase access to chronic NCD care with minimal budget impact in western Kenya. Our budget impact model was highly sensitive to scale‐up and human resource costs, and was influenced to a lesser extent by facility mix and travel costs. The programmatic costing and BIA estimates provide policy makers with a framework to estimate resource needs to expand NCD care within existing health system structures, and the flexibility to integrate care within HIV or primary care platforms based on county‐level capacity.

## 6 Research agenda

This research aims to:

- Present findings from a novel budget impact analysis conducted for a dedicated chronic NCD care programme in East Africa.
- Measure clinic‐level monthly costs of the AMPATH Chronic Disease Management (CDM) programme in western Kenya, which builds on an establish HIV service platform to provide chronic NCD care to more than 15,000 patients.
- Model the budget impact of increasing the number of annual CDM patients to reach 50% of the eligible population in six counties by 2025.
- Vary the provider costs and type of offering facilities to assess the optimal scenarios under which the CDM program can be scale‐up with minimal budget impact.

## 7 Policy Implications and Future Directions

- Scaling up CDM to the target population coverage under a constant annual growth scenario has a lower five‐year budget impact than a steady‐stage enrolment.
- The budget impact of scaling up CDM under to the target patient coverage is $3,088,928 ($2,323,970 to $3,891,191).
- The static model used in this BIA is consistent with ISPOR guidelines, which contributes a reliable and adaptable framework for policy makers to estimate the resources required to layer NCD care onto existing health system structures.
- Task‐shifting chronic NCD care is a highly cost‐saving approach for reaching a wider, rural patient population and one that should be emphasized during scale‐up activities.
- The annual total cost of CDM at the target coverage is less than 1% of each county’s annual health budget.

## Competing interests

Authors have no competing interests to declare.

## Authors’ contributions

BO, AM, JL and OG contributed to conception and design. BO and MWB contributed to analysis. All authors contributed to interpretation and important intellectual input. SP, KR, JK and JL contributed to contextual and medical model development and interpretation. BO and OG contributed to first draft of manuscript. All authors read and approved the final manuscript.

## Supporting information


**Table S1.** Health facility level descriptions, [1]
**Table S2.** Number of health facilities by level and county
**Table S3.** County level annual patient visits
**Table S4.** Percent patient‐visit growth from prior year
**Table S5.** Patient visits
**Table S6.** Clinical staff salary
**Table S7.** Clinical staff training
**Table S8.** Travel costs
**Table S9.** Equipment costs
**Table S10.** Administrative costs
**Table S11.** Budget impact analysis assumptions
**Table S12.** Scale‐up inputs quantifying 2025 patient population goal
**Table S13.** Steady state patient enrolment scale‐up inputs
**Table S14.** Constant percent growth enrolment scale‐up inputs
**Table S15.** Facility mix scenario breakdown for BIA
**Table S16.** Steady state scale‐up under historic distribution of patients across health facility levels
**Table S17.** Steady state scale‐up under primary care weighted distribution of patients across health facility levels
**Table S18.** Steady state scale‐up under equal numbers of patients across health facility levels
**Table S19.** Consistent growth percent scale‐up under historical distribution of patients across health facility levels
**Table S20.** Consistent growth percent scale‐up under primary care emphasis distribution of patients across health facility levels
**Table S21.** Consistent growth percent scale‐up under equal distribution of patients across health facility levels
**Table S22.** Transport costs: consistent growth percent scale‐up under equal distribution of patients across health facility levels
**Table S23.** Transport costs: steady state scale‐up under equal distribution of patients across health facility levels
