# Supplementary material for: Layering and scaling up chronic non‐communicable disease care on existing HIV care systems and acute care settings in Kenya: a cost and budget impact analysis
Source: J Int AIDS Soc. 2020 Jun 19;23(Suppl 1):e25496. doi: 10.1002/jia2.25496 (PMC7305417; doi:10.1002/jia2.25496)
Supplement: Supplementary file 1 — Table S1. Health facility level descriptions, [1] Table S2. Number of health facilities by level and county Table S3. County level annual patient visits Table S4. Percent patient‐visit growth from prior year Table S5. Patient visits Table S6. Clinical staff salary Table S7. Clinical staff training Table S8. Travel costs Table S9. Equipment costs Table S10. Administrative costs Table S11. Budget impact analysis assumptions Table S12. Scale‐up inputs quantifying 2025 patient population goal Table S13. Steady state patient enrolment scale‐up inputs Table S14. Constant percent growth enrolment scale‐up inputs Table S15. Facility mix scenario breakdown for BIA Table S16. Steady state scale‐up under historic distribution of patients across health facility levels Table S17. Steady state scale‐up under primary care weighted distribution of patients across health facility levels Table S18. Steady state scale‐up under equal numbers of patients across health facility levels Table S19. Consistent growth percent scale‐up under historical distribution of patients across health facility levels Table S20. Consistent growth percent scale‐up under primary care emphasis distribution of patients across health facility levels Table S21. Consistent growth percent scale‐up under equal distribution of patients across health facility levels Table S22. Transport costs: consistent growth percent scale‐up under equal distribution of patients across health facility levels Table S23. Transport costs: steady state scale‐up under equal distribution of patients across health facility levels [file JIA2-23-e25496-s001.docx]

**Appendix to *Layering and Scaling-Up Chronic Non-Communicable Disease Care on Existing HIV Care Systems and Acute Care Settings in Kenya: A Cost and Budget Impact Analysis***

Table of Contents

[Health Facility Details 2](#_Toc33741560)

[Table S1: Health Facility Level Descriptions, [1] 2](#_Toc33741561)

[Table S2:Number of health facilities by level and county 3](#_Toc33741562)

[Patient Visit Trends from 2014-2018 3](#_Toc33741563)

[TableS3: County Level Annual Patient Visits 3](#_Toc33741564)

[TableS4: Percent Patient-visit Growth from Prior Year 3](#_Toc33741565)

[Cost Categorizations and Facility Attribution Protocols 4](#_Toc33741566)

[Table S5: Patient Visits 4](#_Toc33741567)

[Table S6: Clinical Staff Salary 4](#_Toc33741568)

[Table S7:Clinical Staff Training: 4](#_Toc33741569)

[Table S8: Travel Costs: 4](#_Toc33741570)

[Table S9: Equipment Costs 4](#_Toc33741571)

[Table S10: Administrative Costs: 4](#_Toc33741572)

[Table S11: Budget Impact Analysis Assumptions 5](#_Toc33741573)

[Patient Inputs and Scale-up Scenarios by County 5](#_Toc33741574)

[Table S12 Scale-up Inputs quantifying 2025 patient population goal 5](#_Toc33741575)

[Table S13: Steady State Patient Enrollment Scale-Up Inputs 5](#_Toc33741576)

[Table S14: Constant Percent Growth Enrollment Scale-Up Inputs 6](#_Toc33741577)

[Table S15: Facility Mix Scenario Breakdown for BIA 6](#_Toc33741578)

[Sensitivity Analysis BIA Results 7](#_Toc33741579)

[Table S16: Steady State Scale-up Under Historic Distribution of patients across Health Facility Levels 7](#_Toc33741580)

[Table S17: Steady State Scale-up Under Primary Care Weighted Distribution of patients across Health Facility Levels 8](#_Toc33741581)

[Table S18: Steady State Scale-Up Under Equal numbers of patients across Health Facility Levels 9](#_Toc33741582)

[Table S19: Consistent Growth Percent Scale-Up Under Historical Distribution of patients across Health Facility Levels 10](#_Toc33741583)

[Table S20: Consistent Growth Percent Scale-Up Under Primary Care Emphasis Distribution of patients across Health Facility Levels 11](#_Toc33741584)

[Table S21: Consistent Growth Percent Scale-Up Under Equal Distribution of patients across Health Facility Levels 12](#_Toc33741585)

[Table S22: Transport Costs: Consistent Growth Percent Scale-Up Under Equal Distribution of patients across Health Facility Levels 13](#_Toc33741586)

[Table S23: Transport Costs: Steady State Scale-Up Under Equal Distribution of patients across Health Facility Levels 14](#_Toc33741587)

[References: 15](#_Toc33741588)

# Health Facility Details

## Table S1: Health Facility Level Descriptions, [1]

| Health Facility Level | Location | Level of Care | Clinical Staff Mix | Catchment Population |
| --- | --- | --- | --- | --- |
| Dispensary | Village Level | Primary Care (static or mobile clinics)  Outpatient services for uncomplicated medical cases. Otherwise refer to Health Center | Typically run and managed by registered nurses and nursing officer. Occasionally includes a clinical officer | 10,000  provide an average of 30 outpatient services (curative, preventive, or heath promotive) per day. |
| Health Centre | Locational Level | Comprehensive Primary Care | Clinical Officer in charge, Nurses, Health Administrator  Possibly: medical techonologist, pharmacist, public Health Officer, nutritionist, and supporting staff. | 30,000  Minimum same as above plus be able to handle at least four deliveries per day. |
| Hospital | District or County | Sub-county or county level referral services | Same as above plus medical officers (physicians), more surgical capacity  More complex patients are referred to higher capacity hospitals as the capacity in this category is varied.  Coordinating and referral center for the smaller units. | Primary referral facilities are targeted to serve a population of 100,000  County Hospitals can serve populations up to 500,000 |
| Referral Hospital  Moi teaching and Referral Hospital, (MTRH) | Eldoret, Uasin Gishu County | National | Regional centers providing specialized care, including intensive care, centers of excellence, and training of clinical officers and medical students.  Highest level of referral systems for the most complex cases. | 3-5 million |

## Table S2:Number of health facilities by level and county

| total | Dispensaries | Health Centers | Primary Hospitals | Referral Hospital | Regional Referral Hospital | All Facilities |
| --- | --- | --- | --- | --- | --- | --- |
| Bungoma | 180 | 37 | 16 | 1 | 0 | 234 |
| Busia | 100 | 29 | 114 | 1 | 0 | 244 |
| Kisumu | 172 | 49 | 36 | 1 | 0 | 258 |
| Nandi | 169 | 21 | 9 | 1 | 0 | 200 |
| Trans Nzoia | 130 | 28 | 13 | 0 | 0 | 171 |
| Uasin Gishu | 165 | 31 | 17 | 0 | 1 | 214 |
| total | 916 | 195 | 205 | 4 | 1 | 1321 |

# Patient Visit Trends from 2014-2018

## TableS3: County Level Annual Patient Visits

|  | 2014 | 2015 | 2016 | 2017 | 2018 |
| --- | --- | --- | --- | --- | --- |
| Bungoma | 1,054 | 1,591 | 3,119 | 1,986 | 2,972 |
| Busia | 2,072 | 8,402 | 11,639 | 12,920 | 7,365 |
| Kisumu | 18 | 685 | 1,419 | 1,333 | 1,070 |
| Nandi | 2,612 | 2,015 | 2,349 | 1,862 | 1,423 |
| TransNzoia | 1,427 | 3,939 | 6,402 | 6,369 | 11,233 |
| UasinGishu | 9,711 | 1,0857 | 13,123 | 13,133 | 16,403 |
| Total | 16,894 | 27,489 | 38,051 | 37,603 | 40466 |

## TableS4: Percent Patient-visit Growth from Prior Year

|  | 2014 | 2015 | 2016 | 2017 | 2018 |
| --- | --- | --- | --- | --- | --- |
| Bungoma | - | 51 | 96 | -36 | 50 |
| Busia | - | 306 | 39 | 11 | -43 |
| Kisumu | - | 3,706 | 107 | -6 | -20 |
| Nandi | - | -23 | 17 | -21 | -24 |
| TransNzoia | - | 176 | 63 | 0 | 76 |
| UasinGishu | - | 12 | 21 | 0 | 25 |
| Total | - | 63 | 38 | -1 | 7.61 |

# Cost Categorizations and Facility Attribution Protocols

Monthly program costs from 2014-2018 were collected from key informant interviews with program officers and clinicians, CDM program records, and AMPATH employee salary scales and standard operating procedures. Mutually exclusive cost categories were personnel costs for clinicians and programmatic staff, travel, and equipment.

## Table S5: Patient Visits

| Number of Patient Visits | Monthly number of patients by facility 2014-2018. | AMPATH Medical Records System Data Report. At the monthly level patient visits and patients are the same but vary over the course of the year due to follow up schedules |
| --- | --- | --- |

## Table S6: Clinical Staff Salary

| CDM program Nurse Salary | Attributable to facility where assigned by percent effort from Clinical assignment logs 2014-2018 | Cost per patients from monthly patients per facility |
| --- | --- | --- |
| CDM Program |  |  |

## Table S7:Clinical Staff Training:

| Nurses Training | Quarterly Training Costs attributed at facility level with patients during the following quarter | Fraction of total cost attributed by number of facility days/quarter |
| --- | --- | --- |
| Pharmacy training |  |  |
| Clinical Officer Training |  |  |

## Table S8: Travel Costs:

| Bungoma | From Webuye County Hospital | Roundtrip kilometers to geocoded facility from Kenya National Facility Data Set * 75 Kenya shillings (ksh).  In 2014 all transport originated at MTRH, over time this decentralized to other county hospital seats listed here |
| --- | --- | --- |
| Busia | From Port Victoria |  |
| Kisumu | From Kisumu County Referral Hospital |  |
| Nandi | From MTRH |  |
| Trans Nzoia | From Kitale County Hospital |  |
| Uasin Gishu | From MTRH |  |

## Table S9: Equipment Costs

| Electronic Sphygnomementer | $120 USD, replace after 2 years of use for standardization | Costs annualized and divided by patients served during standardized lifespan, costs attributed by monthly patient visits |
| --- | --- | --- |
| A1c DCA Vantex | $2375 USD, replace after 7 years for standardization |  |
| Optum Exceed | $25 new, replace after 7 years for standardization |  |

## Table S10: Administrative Costs:

| Administrative staff | Monthly salary divided by total patients that month | Attributed to facility monthly by number of patient visits |
| --- | --- | --- |
| Data Management |  |  |
| Consulting Medical Officer costs | Monthly Salary by percent effort not specifically assigned to facility, divided by total patients that month. |  |

# Table S11: Budget Impact Analysis Assumptions

| Type |  | Range for sensitivity | Notes and Source |
| --- | --- | --- | --- |
| Average annual patient visits | 9  60% monthly  32% every 2 months  8% every 6 months | 2-12 | Workforce needs assessments provided more specific predictions of patient volume variation by month though enrollment programs. After the first year they approximated the rough  [2] |
| Patient Loss to Follow Up | 10% annually | 0-20% annually | [3] |
| Population | See Input Table |  | [4] |
| Population >40 | 18.5% | 17.7 -21.2 | [4] |
| Proportion of population >40 with 10-year CDV risk>30% or with existing CVD | 7.6% | 95%CI: 5.4%-9.9% | [5] |

# Patient Inputs and Scale-up Scenarios by County

## Table S12 Scale-up Inputs quantifying 2025 patient population goal

|  | pop | population over 40 | Significant risk | 50% target | 2018 #s | 2025 growth goal |
| --- | --- | --- | --- | --- | --- | --- |
| Bungoma | 2,087,733 | 386,231 | 28,967 | 14,484 | 1650 | 12,834 |
| Busia | 624,777 | 115,584 | 8,669 | 4,334 | 6523 | NA |
| Kisumu | 1,192,725 | 220,654 | 16,549 | 8,275 | 696 | 7,578 |
| Nandi | 1,072,438 | 198,401 | 14,880 | 7,440 | 1579 | 5,861 |
| Trans Nzoia | 1,166,145 | 215,737 | 16,180 | 8,090 | 4518 | 3,572 |
| Uasin Gishu | 1,273,568 | 235,610 | 17,671 | 8,835 | 9727 | NA |
| total | 7,417,387 | 1,372,217 | 102,916 | 51,458 | 24,693 | 26,765 |

## Table S13: Steady State Patient Enrollment Scale-Up Inputs

|  | Annual New Patients | **Total Patients**  **2021** | **Total Patients**  **2022** | **Total Patients**  **2023** | **Total Patients**  **2024** | **Total Patients**  **2025** |
| --- | --- | --- | --- | --- | --- | --- |
| Bungoma | 2,567 | 4,216 | 6,783 | 9,350 | 11,917 | 14,484 |
| Busia | - | 6,523 | 6,523 | 6,523 | 6,523 | 6,523 |
| Kisumu | 1,516 | 2,212 | 3,728 | 5,243 | 6,759 | 8,275 |
| Nandi | 1,172 | 2,751 | 3,923 | 5,095 | 6,268 | 7,440 |
| Trans Nzoia | 714 | 5,233 | 5,947 | 6,661 | 7,376 | 8,090 |
| Uasin Gishu | - | 9,727 | 9,727 | 9,727 | 9,727 | 9,727 |
| total | 5,353 | 25,764 | 26,834 | 27,905 | 28,975 | 30,046 |

## Table S14: Constant Percent Growth Enrollment Scale-Up Inputs

|  | Annual Percent Growth | **Total Patients**  **2021** | **Total Patients**  **2022** | **Total Patients**  **2023** | **Total Patients**  **2024** | **Total Patients**  **2025** |
| --- | --- | --- | --- | --- | --- | --- |
| Bungoma | 55% | 2,557 | 3,963 | 6,143 | 9,522 | 14,759 |
| Busia | 0% | 6,523 | 6,523 | 6,523 | 6,523 | 6,523 |
| Kisumu | 65% | 1,149 | 1,895 | 3,127 | 5,160 | 8,514 |
| Nandi | 37% | 2,163 | 2,963 | 4,059 | 5,561 | 7,619 |
| Trans Nzoia | 12% | 5,078 | 5,706 | 6,412 | 7,206 | 8,098 |
| Uasin Gishu | 0% | 9,727 | 9,727 | 9,727 | 9,727 | 9,727 |
| total | 28% | 27,196 | 30,778 | 35,992 | 43,699 | 55,239 |

## Table S15: Facility Mix Scenario Breakdown for BIA

|  | Historically Consistent | Primary Care Weighted | Equal Scale-up |
| --- | --- | --- | --- |
| Dispensary | 21% | 35% | 25% |
| Health Center | 24% | 35% | 25% |
| Primary Hospital | 42% | 20% | 25% |
| Referral Hospital | 13% | 10% | 25% |

# Sensitivity Analysis BIA Results

## Table S16: Steady State Scale-up Under Historic Distribution of patients across Health Facility Levels

| County | Year | New Patients | Mean HR costs Dispensary | Mean HR costs  Health Center | Mean HR costs  Primary Hospital | Mean HR costs  MTRH | Total Program  Minimum HR costs | Minimum HR costs Dispensary | Minimum HR costs Health Center | Minimum HR costs PrimaryH ospital | Minimum HR costsMTRH | Total Program  Minimum HR Costs | Maximum HR costs Dispensary | Maximum HR costs  Health Center | Maximum HR costs  Primary Hospital | Maximum HR costs  MTRH | Total Program  MaximumHR costs |
| --- | --- | --- | --- | --- | --- | --- | --- | --- | --- | --- | --- | --- | --- | --- | --- | --- | --- |
| Bungoma | 1 | 2,567 | 10,826 | 7,162 | 22,925 | 4,598 | 45,511 | 8,299 | 4,848 | 20,398 | 3,582 | 37,127 | 13,356 | 7,958 | 25,427 | 7,292 | 54,034 |
| Bungoma | 2 | 2,567 | 21,651 | 14,325 | 45,850 | 9,196 | 91,022 | 16,599 | 9,696 | 40,796 | 7,164 | 74,255 | 26,713 | 15,916 | 50,855 | 14,585 | 108,068 |
| Bungoma | 3 | 2,567 | 32,477 | 21,487 | 68,775 | 13,794 | 136,533 | 24,898 | 14,544 | 61,195 | 10,746 | 111,382 | 40,069 | 23,874 | 76,282 | 21,877 | 162,102 |
| Bungoma | 4 | 2,567 | 43,303 | 28,649 | 91,700 | 18,393 | 182,044 | 33,197 | 19,392 | 81,593 | 14,328 | 148,509 | 53,425 | 31,832 | 101,709 | 29,170 | 216,136 |
| Bungoma | 5 | 2,567 | 54,129 | 35,812 | 114,625 | 22,991 | 227,556 | 41,496 | 24,240 | 101,991 | 17,909 | 185,637 | 66,782 | 39,791 | 127,136 | 36,462 | 270,171 |
| Bungoma | all | 12,834 | 162,386 | 107,435 | 343,874 | 68,972 | 682,667 | 124,489 | 72,719 | 305,973 | 53,728 | 556,910 | 200,345 | 119,372 | 381,409 | 109,387 | 810,512 |
| Kisumu | 1 | 1,516 | 6,401 | 4,694 | 14,499 | 2,715 | 28,309 | 4,908 | 2,860 | 12,189 | 2,115 | 22,072 | 7,893 | 4,697 | 15,158 | 4,306 | 32,054 |
| Kisumu | 2 | 1,516 | 12,801 | 4,227 | 28,997 | 5,430 | 51,455 | 9,817 | 5,720 | 24,378 | 4,230 | 44,145 | 15,786 | 9,393 | 30,317 | 8,612 | 64,108 |
| Kisumu | 3 | 1,516 | 19,200 | 4,227 | 43,496 | 8,146 | 75,068 | 14,725 | 8,580 | 36,566 | 6,345 | 66,217 | 23,679 | 14,090 | 45,475 | 12,918 | 96,163 |
| Kisumu | 4 | 1,516 | 25,600 | 4,227 | 57,994 | 10,861 | 98,682 | 19,634 | 11,440 | 48,755 | 8,460 | 88,289 | 31,572 | 18,786 | 60,633 | 17,225 | 128,217 |
| Kisumu | 5 | 1,516 | 32,000 | 4,227 | 72,493 | 13,576 | 122,295 | 24,542 | 14,300 | 60,944 | 10,575 | 110,362 | 39,466 | 23,483 | 75,792 | 21,531 | 160,271 |
| Kisumu | all | 1,516 | 96,002 | 21,601 | 217,479 | 40,728 | 375,810 | 73,627 | 42,901 | 182,831 | 31,726 | 331,085 | 118,397 | 70,448 | 227,376 | 64,592 | 480,813 |
| Nandi | 1 | 1,172 | 4,950 | 3,672 | 11,320 | 2,100 | 22,043 | 3,796 | 2,216 | 9,467 | 1,636 | 17,114 | 6,103 | 3,636 | 11,763 | 3,331 | 24,834 |
| Nandi | 2 | 1,172 | 9,901 | 6,946 | 21,834 | 4,200 | 42,881 | 7,591 | 4,432 | 18,933 | 3,272 | 34,228 | 12,206 | 7,273 | 23,527 | 6,661 | 49,667 |
| Nandi | 3 | 1,172 | 14,851 | 10,219 | 32,349 | 6,300 | 63,719 | 11,387 | 6,648 | 28,400 | 4,908 | 51,342 | 18,310 | 10,909 | 35,290 | 9,992 | 74,501 |
| Nandi | 4 | 1,172 | 19,802 | 13,492 | 42,863 | 8,400 | 84,557 | 15,182 | 8,864 | 37,867 | 6,544 | 68,457 | 24,413 | 14,546 | 47,054 | 13,322 | 99,335 |
| Nandi | 5 | 1,172 | 24,752 | 16,765 | 53,378 | 10,500 | 105,395 | 18,978 | 11,080 | 47,333 | 8,179 | 85,571 | 30,516 | 18,182 | 58,817 | 16,653 | 124,169 |
| Nandi | all | 5,861 | 74,256 | 51,093 | 161,744 | 31,500 | 318,593 | 56,934 | 33,240 | 141,999 | 24,538 | 256,712 | 91,549 | 54,547 | 176,452 | 49,958 | 372,506 |
| TransNz | 1 | 714 | 3,019 | 2,047 | 6,545 | 1,280 | 12,890 | 2,316 | 1,352 | 5,753 | 997 | 10,419 | 3,721 | 2,218 | 7,152 | 2,029 | 15,121 |
| TransNz | 2 | 714 | 6,039 | 4,043 | 12,936 | 2,559 | 25,578 | 4,633 | 2,705 | 11,506 | 1,994 | 20,837 | 7,442 | 4,436 | 14,305 | 4,059 | 30,242 |
| TransNz | 3 | 714 | 9,059 | 6,040 | 19,328 | 3,839 | 38,266 | 6,949 | 4,057 | 17,259 | 2,990 | 31,256 | 11,164 | 6,654 | 21,457 | 6,088 | 45,363 |
| TransNz | 4 | 714 | 12,079 | 8,036 | 25,719 | 5,119 | 50,953 | 9,266 | 5,410 | 23,012 | 3,987 | 41,674 | 14,885 | 8,872 | 28,610 | 8,118 | 60,484 |
| TransNz | 5 | 714 | 15,099 | 10,033 | 32,111 | 6,398 | 63,641 | 11,582 | 6,762 | 28,765 | 4,984 | 52,093 | 18,606 | 11,090 | 35,762 | 10,147 | 75,606 |
| TransNz | all | 3,572 | 45,295 | 30,200 | 96,638 | 19,195 | 191,328 | 34,747 | 20,286 | 86,294 | 14,952 | 156,279 | 55,818 | 33,269 | 107,287 | 30,442 | 226,817 |

## Table S17: Steady State Scale-up Under Primary Care Weighted Distribution of patients across Health Facility Levels

| County | Year | New Patients | Mean HR costs Dispensary | Mean HR costs  Health Center | Mean HR costs  Primary Hospital | Mean HR costs  MTRH | Total Program  Minimum HR costs | Minimum HR costs Dispensary | Minimum HR costs Health Center | Minimum HR costs PrimaryH ospital | Minimum HR costsMTRH | Total Program  Minimum HR Costs | Maximum HR costs Dispensary | Maximum HR costs  Health Center | Maximum HR costs  Primary Hospital | Maximum HR costs  MTRH | Total Program  MaximumHR costs |
| --- | --- | --- | --- | --- | --- | --- | --- | --- | --- | --- | --- | --- | --- | --- | --- | --- | --- |
| Bungoma | 1 | 2,567 | 18,051 | 9,734 | 18,837 | 3,537 | 50,158 | 13,839 | 6,359 | 17,633 | 2,755 | 40,586 | 22,269 | 10,894 | 20,028 | 5,610 | 58,802 |
| Bungoma | 2 | 2,567 | 36,101 | 19,468 | 37,673 | 7,074 | 100,316 | 27,677 | 12,718 | 35,267 | 5,511 | 81,172 | 44,539 | 21,789 | 40,056 | 11,219 | 117,603 |
| Bungoma | 3 | 2,567 | 54,152 | 29,202 | 56,510 | 10,611 | 150,475 | 41,516 | 19,076 | 52,900 | 8,266 | 121,758 | 66,808 | 32,683 | 60,085 | 16,829 | 176,405 |
| Bungoma | 4 | 2,567 | 72,202 | 38,936 | 75,346 | 14,148 | 200,633 | 55,354 | 25,435 | 70,534 | 11,021 | 162,345 | 89,078 | 43,578 | 80,113 | 22,438 | 235,207 |
| Bungoma | 5 | 2,567 | 90,253 | 48,670 | 94,183 | 17,685 | 250,791 | 69,193 | 31,794 | 88,167 | 13,776 | 202,931 | 111,347 | 54,472 | 100,141 | 28,048 | 294,008 |
| Bungoma | all | 12,834 | 270,759 | 146,009 | 282,549 | 53,055 | 752,373 | 207,579 | 95,382 | 264,502 | 41,329 | 608,792 | 334,041 | 163,417 | 300,423 | 84,144 | 882,025 |
| Kisumu | 1 | 1,516 | 10,656 | 6,427 | 11,656 | 2,089 | 30,827 | 8,168 | 3,752 | 10,556 | 1,627 | 24,103 | 13,145 | 6,430 | 11,970 | 3,312 | 34,858 |
| Kisumu | 2 | 1,516 | 21,310 | 5,745 | 23,312 | 4,177 | 54,545 | 16,336 | 7,504 | 21,112 | 3,254 | 48,206 | 26,289 | 12,861 | 23,941 | 6,625 | 69,716 |
| Kisumu | 3 | 1,516 | 31,965 | 5,745 | 34,968 | 6,266 | 78,944 | 24,504 | 11,257 | 31,669 | 4,881 | 72,310 | 39,434 | 19,291 | 35,911 | 9,937 | 104,574 |
| Kisumu | 4 | 1,516 | 42,619 | 5,745 | 46,624 | 8,354 | 103,343 | 32,671 | 15,009 | 42,225 | 6,508 | 96,413 | 52,579 | 25,722 | 47,881 | 13,250 | 139,432 |
| Kisumu | 5 | 1,516 | 53,273 | 5,745 | 58,280 | 10,443 | 127,742 | 40,839 | 18,761 | 52,781 | 8,135 | 120,516 | 65,724 | 32,152 | 59,851 | 16,562 | 174,289 |
| Kisumu | all | 1,516 | 159,823 | 29,408 | 174,841 | 31,329 | 395,401 | 122,518 | 56,283 | 158,343 | 24,405 | 361,548 | 197,171 | 96,457 | 179,554 | 49,686 | 522,868 |
| Nandi | 1 | 1,172 | 8,247 | 5,030 | 9,086 | 1,615 | 23,979 | 6,323 | 2,906 | 8,204 | 1,258 | 18,691 | 10,171 | 4,978 | 9,298 | 2,562 | 27,008 |
| Nandi | 2 | 1,172 | 16,495 | 9,478 | 17,789 | 3,231 | 46,992 | 12,646 | 5,812 | 16,408 | 2,517 | 37,382 | 20,343 | 9,955 | 18,595 | 5,124 | 54,017 |
| Nandi | 3 | 1,172 | 24,742 | 13,925 | 26,492 | 4,846 | 70,006 | 18,968 | 8,718 | 24,612 | 3,775 | 56,073 | 30,514 | 14,933 | 27,893 | 7,686 | 81,025 |
| Nandi | 4 | 1,172 | 32,990 | 18,373 | 35,195 | 6,462 | 93,019 | 25,291 | 11,624 | 32,816 | 5,034 | 74,765 | 40,685 | 19,910 | 37,191 | 10,248 | 108,034 |
| Nandi | 5 | 1,172 | 41,237 | 22,820 | 43,898 | 8,077 | 116,032 | 31,614 | 14,530 | 41,020 | 6,292 | 93,456 | 50,856 | 24,888 | 46,488 | 12,810 | 135,042 |
| Nandi | all | 5,861 | 123,711 | 69,626 | 132,461 | 24,231 | 350,028 | 94,842 | 43,591 | 123,059 | 18,876 | 280,367 | 152,569 | 74,663 | 139,465 | 38,429 | 405,127 |
| TransNz | 1 | 714 | 5,029 | 2,786 | 5,360 | 984 | 14,160 | 3,858 | 1,773 | 4,983 | 767 | 11,381 | 6,201 | 3,035 | 5,650 | 1,561 | 16,447 |
| TransNz | 2 | 714 | 10,059 | 5,498 | 10,648 | 1,969 | 28,174 | 7,715 | 3,546 | 9,967 | 1,534 | 22,761 | 12,402 | 6,070 | 11,300 | 3,122 | 32,895 |
| TransNz | 3 | 714 | 15,090 | 8,210 | 15,936 | 2,953 | 42,188 | 11,573 | 5,319 | 14,950 | 2,300 | 34,142 | 18,604 | 9,105 | 16,950 | 4,683 | 49,342 |
| TransNz | 4 | 714 | 20,120 | 10,922 | 21,223 | 3,937 | 56,203 | 15,430 | 7,091 | 19,934 | 3,067 | 45,523 | 24,805 | 12,141 | 22,600 | 6,245 | 65,790 |
| TransNz | 5 | 714 | 25,150 | 13,635 | 26,511 | 4,922 | 70,217 | 19,288 | 8,864 | 24,917 | 3,834 | 56,904 | 31,006 | 15,176 | 28,250 | 7,806 | 82,237 |
| TransNz | all | 3,572 | 75,448 | 41,051 | 79,678 | 14,765 | 210,942 | 57,864 | 26,593 | 74,752 | 11,502 | 170,711 | 93,019 | 45,527 | 84,749 | 23,417 | 246,712 |

## Table S18: Steady State Scale-Up Under Equal numbers of patients across Health Facility Levels

| County | Year | New Patients | Mean HR costs Dispensary | Mean HR costs  Health Center | Mean HR costs  Primary Hospital | Mean HR costs  MTRH | Total Program  Minimum HR costs | Minimum HR costs Dispensary | Minimum HR costs Health Center | Minimum HR costs PrimaryH ospital | Minimum HR costsMTRH | Total Program  Minimum HR Costs | Maximum HR costs Dispensary | Maximum HR costs  Health Center | Maximum HR costs  Primary Hospital | Maximum HR costs  MTRH | Total Program  MaximumHR costs |
| --- | --- | --- | --- | --- | --- | --- | --- | --- | --- | --- | --- | --- | --- | --- | --- | --- | --- |
| Bungoma | 1 | 2,567 | 10,826 | 7,162 | 22,925 | 4,598 | 45,511 | 8,299 | 4,848 | 20,398 | 3,582 | 37,127 | 13,356 | 7,958 | 25,427 | 7,292 | 54,034 |
| Bungoma | 2 | 2,567 | 21,651 | 14,325 | 45,850 | 9,196 | 91,022 | 16,599 | 9,696 | 40,796 | 7,164 | 74,255 | 26,713 | 15,916 | 50,855 | 14,585 | 108,068 |
| Bungoma | 3 | 2,567 | 32,477 | 21,487 | 68,775 | 13,794 | 136,533 | 24,898 | 14,544 | 61,195 | 10,746 | 111,382 | 40,069 | 23,874 | 76,282 | 21,877 | 162,102 |
| Bungoma | 4 | 2,567 | 43,303 | 28,649 | 91,700 | 18,393 | 182,044 | 33,197 | 19,392 | 81,593 | 14,328 | 148,509 | 53,425 | 31,832 | 101,709 | 29,170 | 216,136 |
| Bungoma | 5 | 2,567 | 54,129 | 35,812 | 114,625 | 22,991 | 227,556 | 41,496 | 24,240 | 101,991 | 17,909 | 185,637 | 66,782 | 39,791 | 127,136 | 36,462 | 270,171 |
| Bungoma | all | 12,834 | 162,386 | 107,435 | 343,874 | 68,972 | 682,667 | 124,489 | 72,719 | 305,973 | 53,728 | 556,910 | 200,345 | 119,372 | 381,409 | 109,387 | 810,512 |
| Kisumu | 1 | 1,516 | 6,401 | 4,694 | 14,499 | 2,715 | 28,309 | 4,908 | 2,860 | 12,189 | 2,115 | 22,072 | 7,893 | 4,697 | 15,158 | 4,306 | 32,054 |
| Kisumu | 2 | 1,516 | 12,801 | 4,227 | 28,997 | 5,430 | 51,455 | 9,817 | 5,720 | 24,378 | 4,230 | 44,145 | 15,786 | 9,393 | 30,317 | 8,612 | 64,108 |
| Kisumu | 3 | 1,516 | 19,200 | 4,227 | 43,496 | 8,146 | 75,068 | 14,725 | 8,580 | 36,566 | 6,345 | 66,217 | 23,679 | 14,090 | 45,475 | 12,918 | 96,163 |
| Kisumu | 4 | 1,516 | 25,600 | 4,227 | 57,994 | 10,861 | 98,682 | 19,634 | 11,440 | 48,755 | 8,460 | 88,289 | 31,572 | 18,786 | 60,633 | 17,225 | 128,217 |
| Kisumu | 5 | 1,516 | 32,000 | 4,227 | 72,493 | 13,576 | 122,295 | 24,542 | 14,300 | 60,944 | 10,575 | 110,362 | 39,466 | 23,483 | 75,792 | 21,531 | 160,271 |
| Kisumu | all | 1,516 | 96,002 | 21,601 | 217,479 | 40,728 | 375,810 | 73,627 | 42,901 | 182,831 | 31,726 | 331,085 | 118,397 | 70,448 | 227,376 | 64,592 | 480,813 |
| Nandi | 1 | 1,172 | 4,950 | 3,672 | 11,320 | 2,100 | 22,043 | 3,796 | 2,216 | 9,467 | 1,636 | 17,114 | 6,103 | 3,636 | 11,763 | 3,331 | 24,834 |
| Nandi | 2 | 1,172 | 9,901 | 6,946 | 21,834 | 4,200 | 42,881 | 7,591 | 4,432 | 18,933 | 3,272 | 34,228 | 12,206 | 7,273 | 23,527 | 6,661 | 49,667 |
| Nandi | 3 | 1,172 | 14,851 | 10,219 | 32,349 | 6,300 | 63,719 | 11,387 | 6,648 | 28,400 | 4,908 | 51,342 | 18,310 | 10,909 | 35,290 | 9,992 | 74,501 |
| Nandi | 4 | 1,172 | 19,802 | 13,492 | 42,863 | 8,400 | 84,557 | 15,182 | 8,864 | 37,867 | 6,544 | 68,457 | 24,413 | 14,546 | 47,054 | 13,322 | 99,335 |
| Nandi | 5 | 1,172 | 24,752 | 16,765 | 53,378 | 10,500 | 105,395 | 18,978 | 11,080 | 47,333 | 8,179 | 85,571 | 30,516 | 18,182 | 58,817 | 16,653 | 124,169 |
| Nandi | all | 5,861 | 74,256 | 51,093 | 161,744 | 31,500 | 318,593 | 56,934 | 33,240 | 141,999 | 24,538 | 256,712 | 91,549 | 54,547 | 176,452 | 49,958 | 372,506 |
| TransNz | 1 | 714 | 3,019 | 2,047 | 6,545 | 1,280 | 12,890 | 2,316 | 1,352 | 5,753 | 997 | 10,419 | 3,721 | 2,218 | 7,152 | 2,029 | 15,121 |
| TransNz | 2 | 714 | 6,039 | 4,043 | 12,936 | 2,559 | 25,578 | 4,633 | 2,705 | 11,506 | 1,994 | 20,837 | 7,442 | 4,436 | 14,305 | 4,059 | 30,242 |
| TransNz | 3 | 714 | 9,059 | 6,040 | 19,328 | 3,839 | 38,266 | 6,949 | 4,057 | 17,259 | 2,990 | 31,256 | 11,164 | 6,654 | 21,457 | 6,088 | 45,363 |
| TransNz | 4 | 714 | 12,079 | 8,036 | 25,719 | 5,119 | 50,953 | 9,266 | 5,410 | 23,012 | 3,987 | 41,674 | 14,885 | 8,872 | 28,610 | 8,118 | 60,484 |
| TransNz | 5 | 714 | 15,099 | 10,033 | 32,111 | 6,398 | 63,641 | 11,582 | 6,762 | 28,765 | 4,984 | 52,093 | 18,606 | 11,090 | 35,762 | 10,147 | 75,606 |
| TransNz | all | 3,572 | 45,295 | 30,200 | 96,638 | 19,195 | 191,328 | 34,747 | 20,286 | 86,294 | 14,952 | 156,279 | 55,818 | 33,269 | 107,287 | 30,442 | 226,817 |

## Table S19: Consistent Growth Percent Scale-Up Under Historical Distribution of patients across Health Facility Levels

| County | Year | New Patients | Mean HR costs Dispensary | Mean HR costs  Health Center | Mean HR costs  Primary Hospital | Mean HR costs  MTRH | Total Program  Minimum HR costs | Minimum HR costs Dispensary | Minimum HR costs Health Center | Minimum HR costs PrimaryH ospital | Minimum HR costsMTRH | Total Program  Minimum HR Costs | Maximum HR costs Dispensary | Maximum HR costs  Health Center | Maximum HR costs  Primary Hospital | Maximum HR costs  MTRH | Total Program  MaximumHR costs |
| --- | --- | --- | --- | --- | --- | --- | --- | --- | --- | --- | --- | --- | --- | --- | --- | --- | --- |
| Bungoma | 1 | 907 | 3,832 | 2,534 | 8,303 | 1,625 | 16,295 | 2,940 | 1,716 | 7,410 | 1,266 | 13,333 | 4,726 | 2,816 | 9,187 | 2,578 | 19,307 |
| Bungoma | 2 | 1,406 | 9,767 | 6,464 | 20,895 | 4,145 | 41,271 | 7,492 | 4,378 | 18,618 | 3,229 | 33,716 | 12,046 | 7,181 | 23,151 | 6,573 | 48,952 |
| Bungoma | 3 | 2,180 | 18,963 | 12,549 | 40,376 | 8,050 | 79,937 | 14,542 | 8,497 | 35,952 | 6,271 | 65,262 | 23,390 | 13,942 | 44,756 | 12,767 | 94,855 |
| Bungoma | 4 | 3,379 | 33,214 | 21,979 | 70,558 | 14,102 | 139,853 | 25,467 | 14,881 | 62,808 | 10,986 | 114,141 | 40,973 | 24,420 | 78,232 | 22,366 | 165,991 |
| Bungoma | 5 | 5,237 | 55,303 | 36,588 | 117,226 | 23,484 | 232,602 | 42,400 | 24,768 | 104,322 | 18,294 | 189,783 | 68,228 | 40,652 | 130,007 | 37,245 | 276,132 |
| Bungoma | all | 13,109 | 121,079 | 80,114 | 257,358 | 51,407 | 509,957 | 92,841 | 54,239 | 229,110 | 40,045 | 416,235 | 149,364 | 89,010 | 285,334 | 81,529 | 605,236 |
| Kisumu | 1 | 453 | 1,912 | 1,404 | 4,392 | 811 | 8,519 | 1,467 | 857 | 3,702 | 631 | 6,657 | 2,355 | 1,405 | 4,589 | 1,286 | 9,635 |
| Kisumu | 2 | 747 | 5,064 | 2,082 | 11,601 | 2,148 | 20,895 | 3,885 | 2,265 | 9,774 | 1,673 | 17,597 | 6,242 | 3,718 | 12,123 | 3,407 | 25,490 |
| Kisumu | 3 | 1,232 | 10,266 | 3,447 | 23,320 | 4,355 | 41,388 | 7,875 | 4,601 | 19,615 | 3,392 | 35,484 | 12,658 | 7,547 | 24,378 | 6,907 | 51,489 |
| Kisumu | 4 | 2,033 | 18,848 | 5,676 | 42,694 | 7,996 | 75,214 | 14,457 | 8,444 | 35,891 | 6,229 | 65,021 | 23,243 | 13,853 | 44,637 | 12,682 | 94,414 |
| Kisumu | 5 | 3,354 | 33,001 | 9,361 | 74,358 | 14,005 | 30,725 | 25,308 | 14,781 | 62,444 | 10,909 | 113,442 | 40,703 | 24,254 | 77,761 | 22,211 | 164,928 |
| Kisumu | all | 7,818 | 69,092 | 21,970 | 156,365 | 29,315 | 276,741 | 52,991 | 30,948 | 131,426 | 22,836 | 238,202 | 85,200 | 50,777 | 163,488 | 46,492 | 345,957 |
| Nandi | 1 | 584 | 2,468 | 1,838 | 5,652 | 1,046 | 11,005 | 1,893 | 1,113 | 4,729 | 815 | 8,550 | 3,041 | 1,820 | 5,873 | 1,659 | 12,394 |
| Nandi | 2 | 800 | 5,844 | 4,080 | 12,801 | 2,480 | 25,206 | 4,480 | 2,633 | 11,163 | 1,932 | 20,208 | 7,202 | 4,311 | 13,875 | 3,933 | 29,321 |
| Nandi | 3 | 1,096 | 10,466 | 7,144 | 22,588 | 4,444 | 44,641 | 8,023 | 4,708 | 19,969 | 3,462 | 36,161 | 12,902 | 7,714 | 24,829 | 7,047 | 52,493 |
| Nandi | 4 | 1,502 | 16,800 | 11,340 | 35,839 | 7,134 | 71,114 | 12,877 | 7,551 | 31,877 | 5,557 | 57,863 | 20,715 | 12,376 | 39,680 | 11,314 | 84,085 |
| Nandi | 5 | 2,058 | 25,482 | 17,085 | 54,005 | 10,820 | 107,393 | 19,532 | 11,440 | 48,204 | 8,429 | 87,606 | 31,423 | 18,759 | 60,039 | 17,160 | 127,380 |
| Nandi | all | 6,040 | 61,061 | 41,488 | 130,885 | 25,924 | 259,359 | 46,806 | 27,445 | 115,942 | 20,194 | 210,387 | 75,283 | 44,980 | 144,296 | 41,114 | 305,673 |
| TransNz | 1 | 559 | 2,368 | 1,610 | 5,046 | 1,002 | 10,026 | 1,819 | 1,066 | 4,426 | 780 | 8,091 | 2,918 | 1,744 | 5,522 | 1,589 | 11,772 |
| TransNz | 2 | 628 | 5,018 | 3,375 | 10,680 | 2,127 | 21,201 | 3,850 | 2,265 | 9,498 | 1,657 | 17,270 | 6,184 | 3,704 | 11,825 | 3,374 | 25,087 |
| TransNz | 3 | 706 | 8,001 | 5,354 | 17,047 | 3,393 | 33,795 | 6,137 | 3,607 | 15,234 | 2,643 | 27,621 | 9,860 | 5,901 | 18,945 | 5,380 | 40,087 |
| TransNz | 4 | 794 | 11,359 | 7,567 | 24,176 | 4,814 | 47,916 | 8,713 | 5,104 | 21,654 | 3,750 | 39,221 | 13,996 | 8,361 | 26,919 | 7,635 | 56,912 |
| TransNz | 5 | 892 | 15,120 | 10,053 | 32,099 | 6,412 | 63,684 | 11,596 | 6,786 | 28,780 | 4,995 | 52,157 | 18,634 | 11,123 | 35,793 | 10,169 | 75,719 |
| TransNz | all | 3,579 | 41,867 | 27,959 | 89,048 | 17,748 | 176,621 | 32,114 | 18,828 | 79,593 | 13,825 | 144,360 | 51,593 | 30,832 | 99,003 | 28,147 | 209,576 |

## Table S20: Consistent Growth Percent Scale-Up Under Primary Care Emphasis Distribution of patients across Health Facility Levels

| County | Year | New Patients | Mean HR costs Dispensary | Mean HR costs  Health Center | Mean HR costs  Primary Hospital | Mean HR costs  MTRH | Total Program  Minimum HR costs | Minimum HR costs Dispensary | Minimum HR costs Health Center | Minimum HR costs PrimaryH ospital | Minimum HR costsMTRH | Total Program  Minimum HR Costs | Maximum HR costs Dispensary | Maximum HR costs  Health Center | Maximum HR costs  Primary Hospital | Maximum HR costs  MTRH | Total Program  MaximumHR costs |
| --- | --- | --- | --- | --- | --- | --- | --- | --- | --- | --- | --- | --- | --- | --- | --- | --- | --- |
| Bungoma | 1 | 907 | 6,375 | 3,443 | 6,858 | 1,250 | 17,927 | 4,888 | 2,250 | 6,432 | 974 | 14,544 | 7,866 | 3,854 | 7,279 | 1,983 | 20,981 |
| Bungoma | 2 | 1,406 | 16,260 | 8,782 | 17,210 | 3,188 | 45,441 | 12,466 | 5,740 | 16,126 | 2,484 | 36,815 | 20,061 | 9,828 | 18,284 | 5,056 | 53,230 |
| Bungoma | 3 | 2,180 | 31,584 | 17,051 | 33,219 | 6,192 | 88,045 | 24,212 | 11,142 | 31,112 | 4,824 | 71,290 | 38,967 | 19,082 | 35,305 | 9,820 | 103,174 |
| Bungoma | 4 | 3,379 | 55,339 | 29,866 | 58,019 | 10,848 | 154,072 | 42,422 | 19,515 | 54,329 | 8,450 | 124,716 | 68,276 | 33,425 | 61,673 | 17,205 | 180,579 |
| Bungoma | 5 | 5,237 | 92,157 | 49,722 | 96,346 | 18,065 | 256,290 | 70,645 | 32,484 | 90,201 | 14,072 | 207,403 | 113,705 | 55,649 | 102,432 | 28,650 | 300,436 |
| Bungoma | all | 13,109 | 201,716 | 108,864 | 211,651 | 39,544 | 561,775 | 154,633 | 71,131 | 198,200 | 30,804 | 454,767 | 248,875 | 121,839 | 224,973 | 62,714 | 658,401 |
| Kisumu | 1 | 453 | 3,189 | 1,921 | 3,543 | 624 | 9,278 | 2,446 | 1,123 | 3,215 | 486 | 7,270 | 3,930 | 1,923 | 3,637 | 989 | 10,478 |
| Kisumu | 2 | 747 | 8,440 | 2,830 | 9,352 | 1,652 | 22,275 | 6,474 | 2,971 | 8,482 | 1,287 | 19,214 | 10,407 | 5,090 | 9,601 | 2,621 | 27,718 |
| Kisumu | 3 | 1,232 | 17,108 | 4,681 | 18,761 | 3,350 | 43,900 | 13,121 | 6,032 | 16,997 | 2,610 | 38,759 | 21,098 | 10,328 | 19,265 | 5,313 | 56,004 |
| Kisumu | 4 | 2,033 | 31,399 | 7,712 | 34,322 | 6,151 | 79,585 | 24,077 | 11,072 | 31,083 | 4,792 | 71,023 | 38,730 | 18,959 | 35,248 | 9,755 | 102,692 |
| Kisumu | 5 | 3,354 | 54,980 | 12,722 | 59,696 | 10,773 | 138,171 | 42,154 | 19,383 | 54,023 | 8,392 | 123,952 | 67,824 | 33,197 | 61,317 | 17,085 | 179,424 |
| Kisumu | all | 7,818 | 115,117 | 29,866 | 125,675 | 22,550 | 293,208 | 88,271 | 40,581 | 113,800 | 17,566 | 260,218 | 141,989 | 69,497 | 129,067 | 35,763 | 376,316 |
| Nandi | 1 | 584 | 4,106 | 2,515 | 4,540 | 805 | 11,965 | 3,147 | 1,456 | 4,100 | 627 | 9,330 | 5,062 | 2,489 | 4,645 | 1,276 | 13,472 |
| Nandi | 2 | 800 | 9,738 | 5,559 | 10,452 | 1,908 | 27,656 | 7,466 | 3,448 | 9,672 | 1,486 | 22,071 | 12,007 | 5,894 | 10,963 | 3,025 | 31,890 |
| Nandi | 3 | 1,096 | 17,444 | 9,720 | 18,544 | 3,418 | 49,126 | 13,371 | 6,168 | 17,297 | 2,663 | 39,499 | 21,511 | 10,552 | 19,611 | 5,421 | 57,096 |
| Nandi | 4 | 1,502 | 28,007 | 15,422 | 29,474 | 5,488 | 78,390 | 21,469 | 9,895 | 27,588 | 4,275 | 63,226 | 34,540 | 16,932 | 31,303 | 8,703 | 91,479 |
| Nandi | 5 | 2,058 | 42,474 | 23,228 | 44,461 | 8,323 | 118,485 | 32,557 | 14,995 | 41,699 | 6,484 | 95,735 | 52,387 | 25,668 | 47,334 | 13,200 | 138,589 |
| Nandi | all | 6,040 | 101,768 | 56,443 | 107,470 | 19,942 | 285,623 | 78,010 | 35,963 | 100,355 | 15,534 | 229,862 | 125,508 | 61,535 | 113,856 | 31,627 | 332,526 |
| TransNz | 1 | 559 | 3,931 | 2,188 | 4,119 | 771 | 11,008 | 3,014 | 1,395 | 3,824 | 600 | 8,833 | 4,848 | 2,383 | 4,345 | 1,222 | 12,799 |
| TransNz | 2 | 628 | 8,358 | 4,583 | 8,782 | 1,637 | 23,359 | 6,410 | 2,964 | 8,219 | 1,275 | 18,868 | 10,305 | 5,062 | 9,327 | 2,595 | 27,290 |
| TransNz | 3 | 706 | 13,322 | 7,269 | 14,058 | 2,610 | 37,258 | 10,214 | 4,721 | 13,194 | 2,033 | 30,163 | 16,426 | 8,068 | 14,961 | 4,139 | 43,594 |
| TransNz | 4 | 794 | 18,909 | 10,278 | 19,961 | 3,703 | 52,851 | 14,499 | 6,686 | 18,760 | 2,885 | 42,829 | 23,315 | 11,435 | 21,267 | 5,873 | 61,889 |
| TransNz | 5 | 892 | 25,185 | 13,657 | 26,505 | 4,932 | 70,280 | 19,310 | 8,893 | 24,925 | 3,842 | 56,970 | 31,053 | 15,217 | 28,264 | 7,822 | 82,357 |
| TransNz | all | 3,579 | 69,705 | 37,975 | 73,424 | 13,652 | 194,756 | 53,447 | 24,660 | 68,921 | 10,635 | 157,662 | 85,947 | 42,166 | 78,164 | 21,652 | 227,929 |

## Table S21: Consistent Growth Percent Scale-Up Under Equal Distribution of patients across Health Facility Levels

| County | Year | New Patients | Mean HR costs Dispensary | Mean HR costs  Health Center | Mean HR costs  Primary Hospital | Mean HR costs  MTRH | Total Program  Minimum HR costs | Minimum HR costs Dispensary | Minimum HR costs Health Center | Minimum HR costs PrimaryH ospital | Minimum HR costsMTRH | Total Program  Minimum HR Costs | Maximum HR costs Dispensary | Maximum HR costs  Health Center | Maximum HR costs  Primary Hospital | Maximum HR costs  MTRH | Total Program  MaximumHR costs |
| --- | --- | --- | --- | --- | --- | --- | --- | --- | --- | --- | --- | --- | --- | --- | --- | --- | --- |
| Bungoma | 1 | 907 | 4,561 | 2,617 | 7,186 | 3,126 | 17,490 | 3,499 | 1,765 | 6,655 | 2,435 | 14,353 | 5,625 | 2,910 | 7,713 | 4,957 | 21,205 |
| Bungoma | 2 | 1,406 | 11,620 | 6,675 | 18,048 | 7,971 | 44,314 | 8,911 | 4,502 | 16,692 | 6,209 | 36,314 | 14,334 | 7,422 | 19,390 | 12,641 | 53,788 |
| Bungoma | 3 | 2,180 | 22,569 | 12,958 | 34,845 | 15,480 | 85,852 | 17,305 | 8,737 | 32,212 | 12,059 | 70,313 | 27,841 | 14,409 | 37,453 | 24,551 | 104,254 |
| Bungoma | 4 | 3,379 | 39,529 | 22,696 | 60,869 | 27,120 | 150,214 | 30,305 | 15,302 | 56,256 | 21,126 | 122,989 | 48,768 | 25,238 | 65,437 | 43,011 | 182,454 |
| Bungoma | 5 | 5,237 | 65,823 | 37,782 | 101,092 | 45,162 | 249,858 | 50,459 | 25,469 | 93,410 | 35,180 | 204,519 | 81,211 | 42,016 | 108,699 | 71,625 | 303,550 |
| Bungoma | all | 13,109 | 144,102 | 82,727 | 222,039 | 98,859 | 547,727 | 110,479 | 55,775 | 205,225 | 77,009 | 448,488 | 177,779 | 91,995 | 238,691 | 156,786 | 665,251 |
| Kisumu | 1 | 453 | 2,283 | 1,451 | 3,736 | 1,559 | 9,030 | 1,753 | 881 | 3,326 | 1,214 | 7,174 | 2,811 | 1,452 | 3,854 | 2,472 | 10,589 |
| Kisumu | 2 | 747 | 6,035 | 2,150 | 9,864 | 4,131 | 22,179 | 4,631 | 2,329 | 8,776 | 3,218 | 18,954 | 7,438 | 3,843 | 10,174 | 6,552 | 28,007 |
| Kisumu | 3 | 1,232 | 12,225 | 3,559 | 19,797 | 8,375 | 43,956 | 9,378 | 4,731 | 17,592 | 6,524 | 38,225 | 15,073 | 7,800 | 20,427 | 13,282 | 56,582 |
| Kisumu | 4 | 2,033 | 22,438 | 5,861 | 36,225 | 15,378 | 79,901 | 17,209 | 8,683 | 32,176 | 11,979 | 70,047 | 27,672 | 14,317 | 37,381 | 24,388 | 103,758 |
| Kisumu | 5 | 3,354 | 39,287 | 9,667 | 63,029 | 26,932 | 138,914 | 30,127 | 15,200 | 55,937 | 20,979 | 122,243 | 48,458 | 25,067 | 65,054 | 42,713 | 181,292 |
| Kisumu | all | 7,818 | 82,269 | 22,688 | 132,650 | 56,374 | 293,981 | 63,098 | 31,824 | 117,806 | 43,915 | 256,643 | 101,453 | 52,478 | 136,890 | 89,407 | 380,228 |
| Nandi | 1 | 584 | 2,938 | 1,900 | 4,792 | 2,012 | 11,643 | 2,253 | 1,144 | 4,243 | 1,567 | 9,207 | 3,620 | 1,881 | 4,924 | 3,191 | 13,617 |
| Nandi | 2 | 800 | 6,957 | 4,215 | 10,986 | 4,769 | 26,926 | 5,333 | 2,707 | 10,010 | 3,715 | 21,766 | 8,575 | 4,455 | 11,625 | 7,563 | 32,218 |
| Nandi | 3 | 1,096 | 12,466 | 7,378 | 19,463 | 8,546 | 47,853 | 9,557 | 4,841 | 17,904 | 6,657 | 38,959 | 15,368 | 7,972 | 20,797 | 13,553 | 57,690 |
| Nandi | 4 | 1,502 | 20,019 | 11,711 | 30,921 | 13,720 | 76,370 | 15,349 | 7,764 | 28,563 | 10,687 | 62,362 | 24,681 | 12,790 | 33,207 | 21,759 | 92,437 |
| Nandi | 5 | 2,058 | 30,360 | 17,644 | 46,630 | 20,808 | 115,441 | 23,277 | 11,763 | 43,177 | 16,209 | 94,426 | 37,435 | 19,387 | 50,221 | 33,000 | 140,044 |
| Nandi | all | 6,040 | 72,740 | 42,848 | 112,792 | 49,854 | 278,233 | 55,769 | 28,219 | 103,897 | 38,836 | 226,721 | 89,681 | 46,485 | 120,774 | 79,066 | 336,006 |
| TransNz | 1 | 559 | 2,811 | 1,663 | 4,329 | 1,926 | 10,729 | 2,156 | 1,096 | 3,960 | 1,501 | 8,713 | 3,465 | 1,802 | 4,613 | 3,055 | 12,935 |
| TransNz | 2 | 628 | 5,968 | 3,485 | 9,213 | 4,091 | 22,758 | 4,577 | 2,328 | 8,510 | 3,187 | 18,602 | 7,357 | 3,827 | 9,895 | 6,489 | 27,568 |
| TransNz | 3 | 706 | 9,515 | 5,528 | 14,737 | 6,524 | 36,304 | 7,296 | 3,708 | 13,658 | 5,082 | 29,744 | 11,730 | 6,098 | 15,867 | 10,347 | 44,042 |
| TransNz | 4 | 794 | 13,497 | 7,813 | 20,919 | 9,258 | 51,487 | 10,347 | 5,248 | 19,417 | 7,212 | 42,224 | 16,640 | 8,640 | 22,552 | 14,683 | 62,515 |
| TransNz | 5 | 892 | 17,979 | 10,381 | 27,777 | 12,330 | 68,467 | 13,783 | 6,978 | 25,801 | 9,605 | 56,167 | 22,166 | 11,495 | 29,975 | 19,555 | 83,192 |
| TransNz | all | 3,579 | 49,771 | 28,869 | 76,975 | 34,130 | 189,745 | 38,160 | 19,358 | 71,347 | 26,587 | 155,451 | 61,359 | 31,863 | 82,901 | 54,129 | 230,251 |

## Table S22: Transport Costs: Consistent Growth Percent Scale-Up Under Equal Distribution of patients across Health Facility Levels

| County | Year | New Patients | Mean Transport Costs Dispensary | Mean Transport Costs  Health Center | Mean Transport Costs  Primary Hospital | Mean Transport Costs  MTRH | Total Program  Minimum Transport Costs | Minimum TRANSPORT Costs Dispensary | Minimum Transport Costs Health Center | Minimum Transport Costs Primary Hospital | Minimum Transport Costs MTRH | Total Program  Minimum Transport Costs | Maximum Transport Costs Dispensary | Maximum Transport Costs  Health Center | Maximum TRANSPORT Costs  Primary Hospital | Maximum Transport Costs  MTRH | Total Program  Maximum Transport Costs |
| --- | --- | --- | --- | --- | --- | --- | --- | --- | --- | --- | --- | --- | --- | --- | --- | --- | --- |
| Bungoma | 1 | 907 | 3,832 | 2,534 | 8,303 | 1,625 | 15,977 | 3,832 | 2,419 | 8,101 | 1,625 | 15,977 | 3,833 | 2,669 | 8,538 | 1,625 | 16,665 |
| Bungoma | 2 | 1,406 | 9,767 | 6,464 | 20,895 | 4,145 | 40,460 | 9,766 | 6,170 | 20,380 | 4,145 | 40,460 | 9,769 | 6,807 | 21,495 | 4,145 | 42,216 |
| Bungoma | 3 | 2,180 | 18,963 | 12,549 | 40,376 | 8,050 | 78,362 | 18,960 | 11,977 | 39,375 | 8,050 | 78,362 | 18,965 | 13,214 | 41,541 | 8,050 | 81,770 |
| Bungoma | 4 | 3,379 | 33,214 | 21,979 | 70,558 | 14,102 | 137,095 | 33,211 | 20,977 | 68,804 | 14,102 | 137,095 | 33,217 | 23,145 | 72,599 | 14,102 | 143,064 |
| Bungoma | 5 | 5,237 | 55,303 | 36,588 | 117,226 | 23,484 | 228,010 | 55,300 | 34,920 | 114,307 | 23,484 | 228,010 | 55,308 | 38,530 | 120,626 | 23,484 | 237,948 |
| Bungoma | all | 13,109 | 121,079 | 80,114 | 257,358 | 51,407 | 499,904 | 121,069 | 76,462 | 250,967 | 51,407 | 499,904 | 121,092 | 84,366 | 264,799 | 51,407 | 521,663 |
| Kisumu | 1 | 453 | 1,912 | 1,404 | 4,392 | 811 | 7,975 | 1,910 | 1,207 | 4,047 | 811 | 7,975 | 1,914 | 1,541 | 4,632 | 811 | 8,899 |
| Kisumu | 2 | 747 | 5,064 | 2,082 | 11,601 | 2,148 | 19,883 | 5,061 | 1,987 | 10,687 | 2,148 | 19,883 | 5,067 | 2,192 | 12,238 | 2,148 | 21,645 |
| Kisumu | 3 | 1,232 | 10,266 | 3,447 | 23,320 | 4,355 | 39,374 | 10,262 | 3,290 | 21,467 | 4,355 | 39,374 | 10,270 | 3,629 | 24,611 | 4,355 | 42,865 |
| Kisumu | 4 | 2,033 | 18,848 | 5,676 | 42,694 | 7,996 | 71,548 | 18,843 | 5,417 | 39,291 | 7,996 | 71,548 | 18,852 | 5,977 | 45,064 | 7,996 | 77,889 |
| Kisumu | 5 | 3,354 | 33,001 | 9,361 | 74,358 | 14,005 | 124,333 | 32,995 | 8,935 | 68,398 | 14,005 | 124,333 | 33,006 | 9,858 | 78,509 | 14,005 | 135,377 |
| Kisumu | all | 7,818 | 69,092 | 21,970 | 156,365 | 29,315 | 263,113 | 69,072 | 20,836 | 143,890 | 29,315 | 263,113 | 69,108 | 23,199 | 165,053 | 29,315 | 286,675 |
| Nandi | 1 | 584 | 2,468 | 1,838 | 5,652 | 1,046 | 10,251 | 2,466 | 1,565 | 5,174 | 1,046 | 10,251 | 2,470 | 1,987 | 5,912 | 1,046 | 11,415 |
| Nandi | 2 | 800 | 5,844 | 4,080 | 12,801 | 2,480 | 24,347 | 5,839 | 3,705 | 12,323 | 2,480 | 24,347 | 5,847 | 4,347 | 13,061 | 2,480 | 25,735 |
| Nandi | 3 | 1,096 | 10,466 | 7,144 | 22,588 | 4,444 | 43,641 | 10,459 | 6,629 | 22,109 | 4,444 | 43,641 | 10,471 | 7,573 | 22,847 | 4,444 | 45,335 |
| Nandi | 4 | 1,502 | 16,800 | 11,340 | 35,839 | 7,134 | 69,919 | 16,790 | 10,635 | 35,360 | 7,134 | 69,919 | 16,806 | 11,992 | 36,098 | 7,134 | 72,031 |
| Nandi | 5 | 2,058 | 25,482 | 17,085 | 54,005 | 10,820 | 105,934 | 25,469 | 16,117 | 53,527 | 10,820 | 105,934 | 25,489 | 18,042 | 54,265 | 10,820 | 108,616 |
| Nandi | all | 6,040 | 61,061 | 41,488 | 130,885 | 25,924 | 254,091 | 61,022 | 38,651 | 128,493 | 25,924 | 254,091 | 61,083 | 43,942 | 132,183 | 25,924 | 263,131 |
| TransNz | 1 | 559 | 2,368 | 1,610 | 5,046 | 1,002 | 9,720 | 2,367 | 1,499 | 4,852 | 1,002 | 9,720 | 2,370 | 1,748 | 5,287 | 1,002 | 10,407 |
| TransNz | 2 | 628 | 5,018 | 3,375 | 10,680 | 2,127 | 20,812 | 5,015 | 3,184 | 10,486 | 2,127 | 20,812 | 5,021 | 3,606 | 10,921 | 2,127 | 21,676 |
| TransNz | 3 | 706 | 8,001 | 5,354 | 17,047 | 3,393 | 33,314 | 7,995 | 5,073 | 16,853 | 3,393 | 33,314 | 8,005 | 5,690 | 17,289 | 3,393 | 34,376 |
| TransNz | 4 | 794 | 11,359 | 7,567 | 24,176 | 4,814 | 47,332 | 11,350 | 7,185 | 23,983 | 4,814 | 47,332 | 11,364 | 8,020 | 24,418 | 4,814 | 48,617 |
| TransNz | 5 | 892 | 15,120 | 10,053 | 32,099 | 6,412 | 62,984 | 15,109 | 9,558 | 31,906 | 6,412 | 62,984 | 15,127 | 10,638 | 32,341 | 6,412 | 64,519 |
| TransNz | all | 3,579 | 41,867 | 27,959 | 89,048 | 17,748 | 174,163 | 41,835 | 26,500 | 88,079 | 17,748 | 174,163 | 41,888 | 29,703 | 90,257 | 17,748 | 179,595 |

## Table S23: Transport Costs: Steady State Scale-Up Under Equal Distribution of patients across Health Facility Levels

| County | Year | New Patients | Mean Transport Costs Dispensary | Mean Transport Costs  Health Center | Mean Transport Costs  Primary Hospital | Mean Transport Costs  MTRH | Total Program  Minimum Transport Costs | Minimum TRANSPORT Costs Dispensary | Minimum Transport Costs Health Center | Minimum Transport Costs Primary Hospital | Minimum Transport Costs MTRH | Total Program  Minimum Transport Costs | Maximum Transport Costs Dispensary | Maximum Transport Costs  Health Center | Maximum TRANSPORT Costs  Primary Hospital | Maximum Transport Costs  MTRH | Total Program  Maximum Transport Costs |
| --- | --- | --- | --- | --- | --- | --- | --- | --- | --- | --- | --- | --- | --- | --- | --- | --- | --- |
| Bungoma | 1 | 2,567 | 10,826 | 7,162 | 22,925 | 4,598 | 16,665 | 10,825 | 6,836 | 22,353 | 4,598 | 44,612 | 10,827 | 7,543 | 23,590 | 4,598 | 46,558 |
| Bungoma | 2 | 2,567 | 21,651 | 14,325 | 45,850 | 9,196 | 42,216 | 21,650 | 13,671 | 44,707 | 9,196 | 89,224 | 21,653 | 15,085 | 47,181 | 9,196 | 93,116 |
| Bungoma | 3 | 2,567 | 32,477 | 21,487 | 68,775 | 13,794 | 81,770 | 32,475 | 20,507 | 67,060 | 13,794 | 133,836 | 32,480 | 22,628 | 70,771 | 13,794 | 139,674 |
| Bungoma | 4 | 2,567 | 43,303 | 28,649 | 91,700 | 18,393 | 143,064 | 43,300 | 27,343 | 89,413 | 18,393 | 178,448 | 43,306 | 30,171 | 94,362 | 18,393 | 186,231 |
| Bungoma | 5 | 2,567 | 54,129 | 35,812 | 114,625 | 22,991 | 237,948 | 54,125 | 34,178 | 111,766 | 22,991 | 223,061 | 54,133 | 37,713 | 117,952 | 22,991 | 232,789 |
| Bungoma | all | 12,834 | 162,386 | 107,435 | 343,874 | 68,972 | 521,663 | 162,376 | 102,535 | 335,299 | 68,972 | 669,182 | 162,399 | 113,140 | 353,857 | 68,972 | 698,368 |
| Kisumu | 1 | 1,516 | 6,401 | 4,694 | 14,499 | 2,715 | 8,899 | 6,399 | 4,034 | 13,343 | 2,715 | 26,491 | 6,403 | 5,154 | 15,303 | 2,715 | 29,575 |
| Kisumu | 2 | 1,516 | 12,801 | 4,227 | 28,997 | 5,430 | 21,645 | 12,798 | 4,034 | 26,686 | 5,430 | 48,948 | 12,803 | 4,451 | 30,607 | 5,430 | 53,292 |
| Kisumu | 3 | 1,516 | 19,200 | 4,227 | 43,496 | 8,146 | 42,865 | 19,197 | 4,034 | 40,030 | 8,146 | 71,406 | 19,204 | 4,451 | 45,910 | 8,146 | 77,711 |
| Kisumu | 4 | 1,516 | 25,600 | 4,227 | 57,994 | 10,861 | 77,889 | 25,595 | 4,034 | 53,373 | 10,861 | 93,863 | 25,604 | 4,451 | 61,213 | 10,861 | 102,130 |
| Kisumu | 5 | 1,516 | 32,000 | 4,227 | 72,493 | 13,576 | 135,377 | 31,994 | 4,034 | 66,716 | 13,576 | 116,320 | 32,005 | 4,451 | 76,517 | 13,576 | 126,549 |
| Kisumu | all | 1,516 | 96,002 | 21,601 | 217,479 | 40,728 | 286,675 | 95,983 | 20,169 | 200,148 | 40,728 | 357,028 | 96,019 | 22,959 | 229,551 | 40,728 | 389,256 |
| Nandi | 1 | 1,172 | 4,950 | 3,672 | 11,320 | 2,100 | 11,415 | 4,948 | 3,124 | 10,360 | 2,100 | 20,531 | 4,952 | 3,970 | 11,841 | 2,100 | 22,863 |
| Nandi | 2 | 1,172 | 9,901 | 6,946 | 21,834 | 4,200 | 25,735 | 9,896 | 6,248 | 20,874 | 4,200 | 41,217 | 9,904 | 7,417 | 22,355 | 4,200 | 43,876 |
| Nandi | 3 | 1,172 | 14,851 | 10,219 | 32,349 | 6,300 | 45,335 | 14,843 | 9,372 | 31,389 | 6,300 | 61,904 | 14,855 | 10,864 | 32,870 | 6,300 | 64,889 |
| Nandi | 4 | 1,172 | 19,802 | 13,492 | 42,863 | 8,400 | 72,031 | 19,791 | 12,495 | 41,903 | 8,400 | 82,590 | 19,807 | 14,310 | 43,385 | 8,400 | 85,902 |
| Nandi | 5 | 1,172 | 24,752 | 16,765 | 53,378 | 10,500 | 108,616 | 24,739 | 15,619 | 52,418 | 10,500 | 103,276 | 24,759 | 17,757 | 53,899 | 10,500 | 106,916 |
| Nandi | all | 5,861 | 74,256 | 51,093 | 161,744 | 31,500 | 263,131 | 74,217 | 46,858 | 156,944 | 31,500 | 309,519 | 74,277 | 54,319 | 164,350 | 31,500 | 324,446 |
| TransNz | 1 | 714 | 3,019 | 2,047 | 6,545 | 1,280 | 10,407 | 3,018 | 1,906 | 6,297 | 1,280 | 12,500 | 3,020 | 2,223 | 6,853 | 1,280 | 13,376 |
| TransNz | 2 | 714 | 6,039 | 4,043 | 12,936 | 2,559 | 21,676 | 6,035 | 3,811 | 12,689 | 2,559 | 25,094 | 6,042 | 4,326 | 13,245 | 2,559 | 26,171 |
| TransNz | 3 | 714 | 9,059 | 6,040 | 19,328 | 3,839 | 34,376 | 9,053 | 5,717 | 19,080 | 3,839 | 37,688 | 9,063 | 6,428 | 19,636 | 3,839 | 38,967 |
| TransNz | 4 | 714 | 12,079 | 8,036 | 25,719 | 5,119 | 48,617 | 12,070 | 7,622 | 25,472 | 5,119 | 50,283 | 12,085 | 8,530 | 26,028 | 5,119 | 51,762 |
| TransNz | 5 | 714 | 15,099 | 10,033 | 32,111 | 6,398 | 64,519 | 15,088 | 9,528 | 31,863 | 6,398 | 62,877 | 15,106 | 10,633 | 32,419 | 6,398 | 64,557 |
| TransNz | all | 3,572 | 45,295 | 30,200 | 96,638 | 19,195 | 179,595 | 45,264 | 28,584 | 95,400 | 19,195 | 188,442 | 45,316 | 32,140 | 98,181 | 19,195 | 194,833 |

# References:

1. Ministry of Health. Norms and Standard Guidelines for the Health Sector: The Kenya Health Strategic and Investment Plan, 2014-2018 [Http://www.health.go.ke/wp-content/uploads/2015/09/16th october WHO Norms and StandarndsBook.pdf]. 2014.

2. Vedanthan R, Lee DJ, Kamano JH, Herasme OI, Kiptoo P, Tulienge D, et al. Hypertension management in rural western Kenya: a needs-based health workforce estimation model. Hum Resour Health. 2019 Dec 16;17(1):57.

3. Osetinsky B, Genberg BL, Bloomfield GS, Hogan J, Pastakia S, Sang E, et al. Hypertension Control and Retention in Care Among HIV-Infected Patients: The Effects of Co-located HIV and Chronic Noncommunicable Disease Care [Www.jaids.com]. 2019.

4. UNICEF Worldpop, OCHA ROSEA. Kenya - Population projection by County (2009 - 2018) and sub-county 2015 - Humanitarian Data Exchange [Https://data.humdata.org/dataset/kenya-population-projection-by-county-2009-2018-and-subcounty-2015]. 2017.

5. MoH-Kenya. Kenya STEPwise Survey for Non Communicable Diseases Risk Factors 2015 Report. 2015;8–210.
